# Effect of vitamin A, calcium and vitamin D fortification and supplementation on nutritional status of women: an overview of systematic reviews

**DOI:** 10.1186/s13643-020-01501-8

**Published:** 2020-10-27

**Authors:** Eti Rajwar, Shradha S. Parsekar, Bhumika Tumkur Venkatesh, Zinnia Sharma

**Affiliations:** Public Health Evidence South Asia, Prasanna School of Public Health, Manipal Academy of Higher Education, Manipal, India

**Keywords:** Food fortification, Overview of systematic reviews, Supplementation, Women

## Abstract

**Background:**

Micronutrient deficiency affects the health and development of vulnerable population such as children and pregnant women. Measures such as fortification of food and supplementation have been implemented to prevent or control deficiencies related to micronutrients.

**Objective:**

To assess the effect of vitamin A, vitamin D, and calcium fortification and supplementation on nutritional status of women in reproductive age group. To assess the toxicities and adverse events related to intervention.

**Methodology:**

Systematic reviews including RCTs on women of reproductive age group provided with vitamin A, vitamin D, and calcium supplementation or fortified food were included, to report all malnutrition-related outcomes due to deficiency of the abovementioned micronutrients. The Cochrane Database of Systematic Reviews, EPPI Centre, Campbell Collaboration, PubMed, Web of Science, and Scopus were searched electronically for English language publications, until 31 March 2018. Hand searching of the articles was done from the *Journal of Food Science and Technology*. Two independent reviewers selected the systematic reviews, extracted data, and assessed for the quality.

**Results:**

A total of 16 systematic reviews were included in narrative synthesis. Supplementation of vitamin A was reported to result in increased maternal serum retinol concentrations and increased breast milk retinol concentration. It reduced the risk of anemia (Hb < 11 g/dL) and reduced maternal clinical infection. Vitamin D supplementation increased 25-hydroxy vitamin D levels. There was insufficient evidence for the effect on bone mineral density and serum calcium levels. Calcium supplementation did not have any significant effect on body weight, weight gain, and body mass index of the participants.

**Conclusion:**

This overview of systematic reviews reiterates the nutritional importance of vitamin A, vitamin D, and calcium supplementation for the reproductive age women. However, there was no empirical evidence available for fortification of food with vitamin A, vitamin D, and calcium and nutritional benefits of the same for reproductive age women, therefore thrusting upon the need of conducting future quality research, i.e., clinical trials and systematic reviews for food fortification.

**Systematic review registration:**

A priori protocol for this overview of systematic reviews was registered in PROSPERO with registration number CRD42018089403.

## Background

Globally, malnutrition is an important public health concern. It affects health of an individual and has an impact on the economic and social development of a country [1]. Managing all forms of malnutrition is an important constituent of the Sustainable Development Goals [2].

Deficiency of important micronutrients is a form of malnutrition that affects the health and development of vulnerable population, viz. children and pregnant women in the low- and middle-income countries (LMICs). Certain important micronutrient deficiencies that have an adverse effect on health are vitamin A, vitamin D, and calcium [2]. Vitamin A [3] and vitamin D deficiencies (even in countries present in low latitudes) have been reported to be a major concern [4]. Deficiency of vitamin A causes blindness in severe cases [3], and deficiency of vitamin D is a cause of rickets in children and it exacerbates osteopenia, osteoporosis, and bone fractures in adults. Deficiency of vitamin D has also been associated with common cancers and their increased risk: infectious diseases, hypertension, and autoimmune disorders [5]. Deficiency of calcium can cause osteoporosis, rickets, and decreased bone mineralization. Although Ca micronutrient deficiency is said to be widespread globally, it is difficult to estimate global burden of the same [6].

Food fortification is one of the most rewarding and widely implemented public health measures to improve the nutritional components of food [7]. Fortification of food as defined by the World Health Organization (WHO) and the Food and Agricultural Organization (FAO) of the United Nations is “the practice of deliberately increasing the content of an essential micronutrient, i.e. vitamins and minerals (including trace elements) in a food, so as to improve the nutritional quality of the food supply and provide a public health benefit with minimal risk to health” [7].

Food fortification is classified into three different types [8]:

- In mass fortification, food that is consumed by a large population is fortified.
- Targeted fortification includes fortifying food that is consumed by a specific target population that is in need.
- In market-driven fortification, the food industry fortifies food based on the regulatory guidelines set by the government.

Fortification of food and supplementation are found to be beneficial especially for reproductive age women, as nutritional status of women has a direct effect on the pregnancy and infant health outcomes. Therefore, these interventions are promoted by the WHO, especially in LMICs. Nevertheless, there is not enough clarity about the implementation of the ideal dose, duration, and timing of these nutritional interventions separately for adolescents, pregnant women, and postpartum women. Additionally, specifically for vitamin D, there are different guidelines for every country/region that makes it difficult to compare the effectiveness of intervention [9].

Although a cost-effective and acceptable strategy for controlling malnutrition [7], food fortification is neither implemented at a larger scale by countries (except for few micronutrients, viz. iodine) nor is its effectiveness being assessed often, therefore leading to a void in the pool of evidence. Considering this, it is imperative to summarize the effect of these interventions on nutritional status of women. Over the years, many systematic reviews (SRs) have been published to examine the effect of vitamin A, vitamin D, and calcium fortification and/or supplementation on health conditions of the vulnerable population. Policy- and decision-makers may face difficulty in accessing the reviews from various sources. Overview of systematic reviews, also called as umbrella reviews, is a coherent mode of gathering the best available evidence of effectiveness of intervention in a single document.

This overview could give a better picture as this presents critically analyzed information of results and evidence from published systematic reviews. The main intention of this overview is to provide the essential information that is required to make a decision to implement the fortification at large scale at public health level to implement nutrition programs and for food managers working at food industries. This implementation could play a major role to draw conclusions on fortification program.

Therefore, in the present overview of systematic reviews, we intend to summarize the available evidence on the effect of vitamin A, vitamin D, and calcium fortification and supplementation on the nutritional status of reproductive age women. Additionally, we will summarize the toxicities and adverse events related to vitamin A, vitamin D, and calcium fortification and supplementation. Our overview will help in making strong evidence available that informs policymakers to make necessary decisions. Subsequently, it will help in advocating and framing the suitable policy on interventions that is more appropriate for the vulnerable population.

## Methodology

An overview of SRs was undertaken by following Cochrane Handbook of Systematic Reviews [10, 11]. We have adhered to the “Preferred Reporting Items of Systematic Reviews and Meta-analysis” [12, 13] to report this overview of SRs; the checklist can be found in Additional file 1. A priori protocol was registered in PROSPERO with registration number CRD42018089403. In the current overview, we have limited the focus on three micronutrients, i.e., vitamin A, vitamin D, and calcium; however, the protocol that was registered in PROSPERO encapsulates multiple micronutrients. Furthermore, to make the paper more concise and specific, effect of micronutrients on the birth outcomes was beyond the scope of this evidence summary. We will publish the evidence of other micronutrients and birth outcomes in subsequent publication.

### Criteria for including SRs for this evidence summary

#### Types of SRs

SRs having a systematic search strategy and covering randomized controlled trials (RCTs) were included.

#### Types of participants

Women of reproductive age group (15–49 years) were included; however, those with life-threatening or serious illness influencing absorption of nutrients were excluded. In case of mixed population, information about participants of our interest was extracted, and if it was not distinctively given, such SRs were excluded.

#### Types of interventions

Included are interventions, in which any mode and vehicle of fortification (except bio-fortification) were used to fortify food with vitamin A, vitamin D, and calcium, either individually or in combination. Additionally, oral supplementation of vitamin A, vitamin D, or calcium, and combination of both fortification and supplementation were eligible for inclusion.

#### Types of comparisons

The following comparisons were considered:

- Supplementation or food fortification (vitamin A, vitamin D, or calcium) vs. control
- Combination of supplementation and fortified food vs. control
- Supplementation along with other micronutrients vs. control

#### Types of outcomes

All nutrition-related outcomes associated with poor intake of vitamin A, vitamin D, and calcium in reproductive age women were considered as primary outcome. Outcomes such as serum retinol levels in women, breast milk retinol concentration, hemoglobin levels, serum calcium levels, serum vitamin D levels, and body weight were included. Any adverse effect reported in the SRs and toxicity associated with fortification and supplementation were considered as secondary outcomes.

### Search methods for identification of SRs

The Cochrane Database of Systematic Reviews, EPPI Centre, Campbell Collaboration, Medline (PubMed), Scopus, and Web of Science were searched electronically for English language publications until 31 March 2018. Hand searching was performed for the *Journal of Food Science and Technology*. References of the included SRs were scanned through for any additional eligible records. Keywords such as “food fortificants,” “supplements,” “women,” and “systematic review” were used to initiate the search. The complete search strategy is present in Additional file 2. The citation yield was exported to EndNote X7, and duplicates were removed. The remaining citations were exported to Excel spreadsheet.

### Selecting eligible SRs

Three reviewers (ER, SSP, ZS) independently assessed the titles and abstracts of SRs for inclusion in two groups. The full-text articles were then assessed by three reviewers (ER, SSP, BTV) independently in groups. Disagreements during the entire selection process were resolved by discussion within and between groups. Screening for SRs was performed by using Excel spreadsheets.

### Data extraction

Extraction of the relevant information was undertaken by three reviewers (ER, SSP, ZS), independently. A predesigned data extraction form consisting of details on participants, interventions, outcomes, study designs, and quality of the included studies was used. Disagreements were resolved via discussion.

### Methodological quality

Quality assessment for the included SRs was performed using “Revised Assessment of Multiple Systematic Reviews” (R-AMSTAR) measurement tool [14, 15], with scoring criteria from 11–44. According to this tool, SRs were marked as high quality if the scores were 34–44, as moderate quality if the scores were 23–33, and as low quality if the scores were 11–22. Methodological quality was assessed independently by ER, SSP, and ZS in groups, and any discrepancies have been resolved by discussion. Data extraction and quality assessment were undertaken on Microsoft Excel spreadsheets.

### Data synthesis

Data synthesis was performed by three review authors (ER, SSP, BTV), and data were summarized narratively. The findings from each SR were thoroughly discussed by authors and are presented in tables. If an outcome was assessed by more than one SR, the result was summarized considering the conclusions of both SRs. If meta-analysis was performed by SRs, then we concluded the findings based on the effect measures. Results of meta-analysis were reported as follows: results for the dichotomous data were presented as summary risk ratio and 95% confidence interval, and for continuous data, mean difference and standardized mean difference were reported.

## Results

**Fig. 1:**
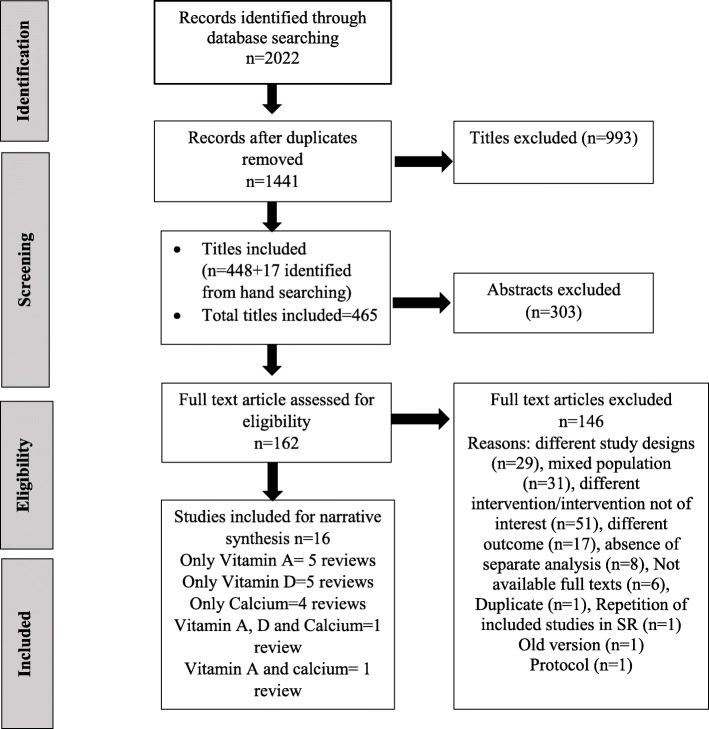
PRISMA flow diagram

The literature search strategy generated 2022 citations; out of these, 581 duplicates were removed. The remaining citations were subjected to title and abstract screening, of which 162 abstracts were included, and finally, 16 SRs were included for evidence synthesis. The details of the selection process and reasons for full-text exclusion are shown in Fig. 1.

Duplicate publication or overlap of information: Palacios et al. [16] and De-Regil et al. [17] are duplicate publications. Hence, we decided to include only De-Regil et al. [17] considering it being the most comprehensive and had scored high on R-AMSTAR. Arthur et al. [18] included one RCT, i.e., Tchum 2006 (list of included RCTs are provided in Additional File 6), for assessing effect of vitamin A supplementation on the serum retinol levels among postpartum women, because it had already been included by Caminha et al. [19]; therefore, Arthur et al. [18] was not included for data synthesis of serum retinol levels. Similarly, one RCT (Martins et al. 2010) of Neves et al. [20] was included by Oliveira et al. [21]; hence, Neves et al. [20] was not considered for synthesis of effect of vitamin A on the serum retinol levels in women. We did not consider two SRs [22, 23], for synthesizing the effect of vitamin D supplementation on serum 25(OH) levels, as included studies of these SRs were covered by Roth et al. [9]. Hence, Perez-Lopez et al. [23] was excluded from the current overview but Christesen et al. [22] was included for another outcome.

### Description of included reviews

The detailed characteristics of the included SRs are mentioned in Additional file 3. However, we have categorized SRs based on the intervention assessed and the summary of characteristics based on the PICO of our interest is described in Table 1.

**Table 1:** Summary characteristics of included systematic review

	Vitamin A	Vitamin D	Calcium
Number of SR	Five SRs (Oliveira et al. [21]; Caminha et al. [19]; Neves et al. [20]; McCauley et al. [24]; Thorne-Lyman and Fawzi [25])	Six SRs (Chakhtoura et al. [26]; Christesen et al. [22]; Reid et al. [27]; De-Regil et al. [17]; Roth et al. [9]; Das et al. [28])	Supplementation: six SRs (Trowman et al. [29]; Cumming [30]; Buppasiri et al. [31]; Onakpoya et al. [32]; Arthur et al. [18]) Fortification: one SR (Das et al. [28])
Population details	Postpartum women, breastfeeding or not, from the region where vitamin A deficiency (VAD) is a major concern, i.e. low-income settings in India, Bangladesh, Indonesia, Tanzania, Gambia, Zimbabwe, Kenya, Ghana, Peru, and Brazil (Oliveira et al. [21]) Pregnant and puerperal women (Neves et al. [20]) Lactating women (Caminha et al. [19]) Pregnant women (Thorne-Lyman and Fawzi [25]; McCauley et al. [24])	Pregnant women (Christesen et al. [22]; De-Regil et al. [17]; Roth et al. [9]) Adult women of age > 18 years (Reid et al. [27]) Pregnant women, children, and adolescents (Chakhtoura et al. [26]) Children, adolescents, pre-pregnant women, women of reproductive age group, and post-menopausal women (Das et al. [28])	Adult women of age > 18 years (Trowman et al. [29]; Cumming [30]) Pregnant women (Buppasiri et al. [31]). Obese/ overweight participants (Onakpoya et al. [32]) Pregnant and puerperal women (Arthur et al. [18]) Pre-pregnant women, women of reproductive age group, and post-menopausal women (Das et al. [28]) included
Intervention	Supplementation of vitamin A alone or in combination with other micronutrients (Oliveira et al. [21]; McCauley et al. [24]; Neves et al. [20]) Supplementation of vitamin A/beta carotene (Thorne-Lyman and Fawzi [25]; Caminha et al. [19])	Supplementation of vitamin D (Chakhtoura et al. [26]; Christesen et al. [22]; Reid et al. [27]; De-Regil et al. [17]; Roth et al. [9]) Fortified food with vitamin D along with other micronutrients (Das et al. [28])	Calcium supplementation with or without dairy products or combined calcium supplementation (Trowman et al. [29]; Cumming [31]; Buppasiri et al. [31]; Onakpoya et al. [32]; Arthur et al. [18]) Alkaline phosphatase and serum parathyroid hormone as a fortificant (Das et al. [28])
Outcomes of our interest	The five included SRs provided information on the effect of vitamin A supplementation on serum retinol levels (Oliveira et al. [21]; Caminha et al. [19]) and hepatic reserves (Oliveira et al. [21]) of the women, vitamin A contents in breast milk (Oliveira et al. [21]; Caminha et al. [19]; Neves et al. [20]), proportion of women with low vitamin A contents (Oliveira et al. [21]), secretary immunoglobulin A levels in colostrum (Neves et al. [20]), subclinical vitamin A deficiency (Oliveira et al. [21]; McCauley et al. [24]), and maternal anemia and hemoglobin (Thorne-Lyman and Fawzi [25])	Vitamin D supplementation: 25(OH)D levels at term/delivery (De-Regil et al. [17]; Roth et al. [9]; Chakhtoura et al. [26]), vitamin D symptoms (Christesen et al. [22]), bone mineral density (Reid et al. [27]), serum calcium levels (Chakhtoura et al. [26]), weight gain (Christesen et al. [22]); side effects of vitamin D (De-Regil et al. [17]), and hypercalcemia, hypocalcemia, hypercalciuria (Roth et al. [9]) Vitamin D fortification: serum vitamin D levels, serum calcium levels (Das et al. [28]) Vitamin D and calcium fortification: serum vitamin D levels, CTx and P1NP (bone resorption marker) levels (Das et al. [28])	The SRs assessed effect of supplementation on body weight, weight gain, BMI, body fat, bone mass, and anemia
Methodological quality (the detail scoring is provided in Additional file 4)	High quality (McCauley et al. [24]; Oliveira et al. [26]) Moderate quality (Neves et al. [20]; Thorne-Lyman and Fawzi [25]) Low quality (Caminha et al. [19])	High quality (De-Regil et al. [17]; Roth et al. [9]) Moderate quality (Chakhtoura et al. [26]; Christesen et al. [22]; Das et al. [28]; Reid et al. [27])	High quality (Buppasiri et al. [31]) Moderate quality (Trowman et al. [29]; Onakpoya et al. [32]; Das et al. [28]) Low quality (Cumming [30]; Arthur et al. [18])

### Effects of vitamin A supplementation and fortification

Five SRs [19–21, 24, 25] provided information on the effect of vitamin A supplementation on nutritional outcomes. Summary of the synthesis is given below, and detailed synthesis is provided in Additional file 5 under table 1.

Vitamin A 200,000–400,000 IU given to women after childbirth increases the *serum retinol levels* when assessed at 3–3.5 months postpartum. Administration of 400,000 IU of vitamin A up to 96 h after childbirth increases the serum retinol levels in mothers, when assessed at 45 days postpartum. However, there is no additional effect on the serum retinol levels by 400,000 IU vitamin A, assessed at 2–6 months, when compared to a standard dose of 200,000 IU. Dosage of 200,000–300,000 IU of vitamin A given to postpartum women increases the serum retinol levels till 6 to 6.5 months postpartum, but the effect was lost when assessed at 9 months postpartum [19, 21].

No substantial increase in *vitamin A hepatic reserves* of postpartum women was reported when supplemented with 200,000–300,000 IU vitamin A for 3, 6, and 9 months after childbirth. Additionally, no significant increase in hepatic reserves was noted with daily supplementation of 7.8 mg beta carotene to postpartum women for 9 months [21].

Vitamin A 200,000 IU given after 12–42 h of childbirth to the postpartum women increases the *breast milk retinol levels*, when assessed from 6 h to 6 months postpartum. Administration of high doses of 200,000–300,000 IU and 200,000–400,000 IU vitamin A increases the retinol levels in breast milk till 3–3.5 months postpartum. However, this effect is not maintained till 6 or 9 months postpartum. The evidence regarding the effect of a single high dose of 400,000 IU vitamin A after delivery and 7.8 mg beta carotene daily for 9 months on breast milk retinol levels was inconclusive. There was no additive effect of a 400,000 IU on the retinol levels in breast milk at 2–4 months, when compared to a standard dose of 200,000 IU after childbirth [19–21]. Vitamin A 200,000–300,000 IU after childbirth reduces the *proportion of women with low retinol levels in breast milk*, in the population assessed at 3 months [21].

Administration of 7000 μg of retinol to pregnant women reduced the *risk of night blindness* [24]. Vitamin A supplementation to both non-anemic and anemic pregnant women reduces the *risk of anemia* [25]; however, there is no effect of vitamin A on *hemoglobin levels* when assessed at 4 to 6 months postpartum [25]. We cannot conclude the effect of vitamin A supplementation on *abnormal conjunctival impression cytology* [21] and *secretary immunoglobulin A* (*sIgA*) *levels in colostrum*, owing to inclusion of lone RCT in the SRs [20].

### Effect of vitamin D alone or vitamin D and calcium combined supplementation and fortification

Six SRs were included to generate the evidence of vitamin D alone or vitamin D and calcium combined [9, 17, 22, 26–28] on nutritional outcomes. Summary of main findings of these outcomes is discussed below, and detailed analysis is provided in Additional file 5.

#### Vitamin D supplementation on nutritional outcomes

*Mean serum 25(OH)D levels* were the most widely reported outcome among pregnant women at full term or childbirth when supplemented with vitamin D [9, 17, 26]. Meta-analysis was undertaken in all three SRs, but sensitivity analysis [9, 26] and sub-group analysis [9, 17] were additionally performed (see Additional file 5). Mean 25(OH)D levels were higher among groups supplemented with vitamin D when compared to no supplementation or placebo group. Additionally, higher dose of vitamin D (≥ 2000 IU/day) and daily dose had better effect than intermediate, or low dose or single dose as studied by three SRs [9, 17, 26]. Although the result was in favor of intervention in all SRs, there were high statistical and clinical heterogeneity and variations in methodological quality of the included RCTs. Based on the GRADE assessment, there is low level of evidence for vitamin D supplementation on 25(OH)D concentration level when compared to no intervention or placebo [17].

There was non-significant result achieved on performing meta-analysis of high vs. intermediate dose and intermediate vs. low dose of vitamin D on *serum calcium levels* [26]. There is insufficient evidence of effect of vitamin D supplementation on *vitamin D deficiency symptoms* [22], *bone mineral density* (*BMD*) [27], *weight gain* [22], and *adverse effects of interventio*n (nephritic syndrome) [17] during pregnancy considering single RCT inclusion in each of the SRs. There was no significant result of vitamin D supplementation on *hypercalcemia* and *hypercalciuria*, while intervention played protective effect on *hypocalcemia* [9].

#### Vitamin D fortification on nutritional outcomes

There is insufficient information about the effect of fortification of vitamin D on *serum vitamin D levels* and *serum calcium levels* as there was only one included RCT for both the outcomes [28].

#### Vitamin D and calcium fortification on nutritional outcomes

There is insufficient information of vitamin D and calcium fortification on *serum vitamin D levels* and bone resorption marker (P1NP and CTX) levels as there was only one included RCT for each outcome [28].

### Effect of calcium supplementation and fortification

To assess the effect of calcium on nutritional outcomes, six SRs [18, 28–32] were included. Summary of main findings of these outcomes is discussed below, and detailed analysis is provided in Additional file 5.

Overall, the evidence suggests that supplementation of calcium (1000 mg/day) for minimum duration of 6 months significantly resulted in weight loss in obese and overweight non-pregnant women when compared to placebo. However, there was no effect of calcium supplementation (1000–2000 mg/ day) as compared to placebo on weight gain during pregnancy [29, 31, 32]. There was not much information to conclude on the effect of calcium supplementation on *bone mass or BMD* [18, 30, 31], *maternal anemia* [30], and *adverse effects of intervention* (maternal cholestasis jaundice, gastrointestinal symptoms like diarrhea, nausea and heartburn, gall stones, and multiple symptoms) considering lone RCT inclusion for each outcome assessment [31].

## Discussion

The overview identified no SRs on the effect of vitamin A fortification on the nutritional outcomes among reproductive age women. Nevertheless, we identified one SR [28] assessing the effect of vitamin D alone or combined with calcium fortification on serum vitamin D levels, serum calcium levels, and bone resorption markers. However, the effect of food fortification is inclusive as there was lone RCT included for each outcome. Therefore, high-quality clinical trials and SRs on fortification of food in women of reproductive age are needed. One of the reasons for lack of evidence on the effect of food fortification can be that the scope of our overview of SRs was only restricted to SRs including RCTs and not to other study designs. The understanding of choice and quantity of fortificant is very crucial for food fortification, e.g., retinyl palmitate and retinyl acetate are the best fortificant choices for the unstable vitamin A; calcium if added in large quantities, as a fortificant, can lead to decreased absorption of iron [7]. Such high technique sensitivity of the process can be a reason for less number of fortification programs. This is often supplemented with poor impact evaluations for such fortification programs, as impact evaluations can be complex and not very cost-effective, leading to low research evidence regarding the effectiveness of these programs.

Additionally, we wanted to summarize the toxicities and adverse events related to nutritional interventions. However, this result is not sufficient for conclusion due to non-availability of adequate evidence as the included SRs' result was based on single RCTs. Fifteen SRs identified the intended effect on study outcomes for reproductive age women, but there was lack of evidence for this age group other than pregnant and postpartum women. Future research can be prioritized in this area.

For vitamin A, the recommended dietary allowance (RDA) is 700–900 μg retinol activity equivalents (RAE) per day and the Tolerable Upper Intake level for adults is 3000 μg of pre-formed vitamin A, per day [33]. The WHO recommends a dose of 10,000 IU of vitamin A per day and 25,000 IU of vitamin A per week, for pregnant women, in areas where vitamin A deficiency is a public health concern [34].

According to our overview of SRs, administration of 200,000–400,000 IU of vitamin A to women post-childbirth can increase the serum retinol levels of women till 3–3.5 months after childbirth. But the effect was lost when follow-up period was increased to 6 months. Previous evidence suggests that serum retinol levels vary during different reproductive age periods and are specifically altered during pregnancy and postpartum period. The levels of vitamin A or serum retinol levels decrease drastically during the third trimester of pregnancy and increase thereafter during the postpartum period. Factors such as hemodilution and nutritional status are supposed to be the reason behind this effect [35]. Therefore, the results identified suggest that the beneficial effect of intervention might be due to the aforementioned factors and might not be specifically due to the intervention. There is a need to control these factors while undertaking the study.

Administration of vitamin A during pregnancy is needed to maintain the serum retinol levels of women residing in areas where vitamin A deficiency is a public health concern, viz. developing and underdeveloped regions of South Asia and Africa [34], but we could not identify a strong evidence of vitamin A supplementation during pregnancy. Serum retinol levels provide information about vitamin A reserves of the liver, only in case of severe depletion, i.e., < 0.07 μmol/g liver, or when extremely high, i.e., > 1.05 μmol/g. Therefore, serum retinol levels are not considered as an ideal indicator of vitamin A status for the individuals; however, distribution of serum retinol levels in a population or the proportion of people having serum retinol levels below the minimum cutoff can be taken as an indicator for status of vitamin A in the entire population, which indicates the extent of vitamin A deficiency as a public health problem [36].

The overview concludes that administration of 200,000 IU vitamin A given after 12–42 h of delivery to the postpartum women increases the breast milk retinol levels. However, this finding cannot be generalized as the results are heterogeneous due to differences in timings and collection of breast milk and baseline vitamin A levels, and sample size in the included RCTs was small and RCTs were from Brazil. It was reported that administration of high doses of vitamin A, viz. 200,000–300,000 IU and 200,000–400,000 IU after childbirth, increases the breast milk retinol levels, but the quality of evidence was low when assessed on the GRADE tool. High breast milk retinol or vitamin A content is indirectly responsible for increasing the vitamin A levels in young children through breast milk [37]. Around 80% of the vitamin A required by the child in the first 2 years of life is contributed by breast milk [38]. Therefore, to avoid deficiency of vitamin A in children, mothers who are breastfeeding should not have vitamin A deficiency.

One SR [24] reported reduction in the risk of night blindness after administration of 7000 μg retinol equivalents to pregnant women in VAD regions of Bangladesh and Nepal. These findings conform with the WHO guidelines that recommend vitamin A administration to pregnant women for preventing night blindness only in VAD regions [34].

Vitamin A has beneficial effect on the risk of anemia on anemic and non-anemic pregnant women (additional iron and folate supplementation was also given to the women), but the result was not significant on severe anemia. More high-quality RCTs on the effect of vitamin A supplementation on maternal anemia are needed with emphasis on the baseline hemoglobin levels of the pregnant women, the technique used for assessment of hemoglobin concentration, and the prevalence of conditions that might alter the hemoglobin levels such as HIV infection/tuberculosis.

Mean serum 25(OH)D levels were the most widely reported outcome among pregnant women at full term or childbirth when supplemented with vitamin D. Supplementation with vitamin D was effective in increasing serum 25(OH)D levels compared to control group. Additionally, higher dose of vitamin D (≥ 2000 IU/day) and daily dose had better effect than intermediate or low dose or single dose. Only one SR [17] performed GRADE for 25(OH)D concentrations and concluded that there is low level of evidence of vitamin D supplementation compared to those who received no intervention or placebo. Although the result was in favor of intervention, there was high statistical heterogeneity related to intervention, outcome assessment, and methodological quality. Additionally, sample size of included trials was small, and confounding factors were not controlled. One SR majorly covered most of the RCTs [9], *n* = 32, which were included by other SRs; hence, there is possibility of overlap of information. Owing to the aforementioned reasons, it is difficult to make a recommendation for clinical practice to use vitamin D supplementation to improve serum 25(OH)D levels in routine antenatal care.

The evidence for effect of vitamin D supplementation on vitamin D deficiency symptoms, BMD, and weight gain was insufficient to conclude, and the effect was not significant on serum calcium levels and side effects of vitamin D.

Majority (approx. 80%) of vitamin D requirement of the body comes from dermal synthesis once sun’s ultraviolet B rays hit the skin, followed by dietary intake (20%) [39, 40]. Cutaneous vitamin D synthesis is affected by season, latitude, time of day, use of sunscreen, aging, and skin pigmentation [39, 40]. Furthermore, naturally, few foods (oily fish, egg yolk) contain vitamin D, which is not consumed by many individuals, e.g., South Asian and vegans. Also, even in tropical countries, despite abundance of sunlight, individuals do not get enough exposure to the sun due to their lifestyle and culture (full body covered clothes) [39]. Dietary consumption of vitamin D, sun exposure, and baseline serum 25(OH)D levels may have confounding effect on serum 25(OH)D concentration; therefore, further studies should take these factors into consideration. Further research is needed where diversity in food should be considered using mathematical modeling to generate evidence [41].

It is recommended to have serum 25(OH)D levels lying between ≥ 25 and ≥ 50 nmol/L (≥ 10 to ≥ 20 ng/mL), which is translated as vitamin D dietary intake of 10–20 μg, i.e., 400–800 IU/day. However, literature suggests low levels of serum 25(OH)D and insufficient dietary intake of vitamin D are prevalent in general population throughout the world, thus an important public health issue. This prevalence is higher in LMICs. Evidence generated from epidemiological studies has demonstrated the effect of low serum 25(OH)D levels on morbidity and mortality. Additionally, deficient 25(OH)D levels may affect adverse pregnancy-related complications and infant outcomes [40]. Need for public health strategies to target the vitamin D levels arises when the proportion of vitamin D-deficient individuals exceeds 2.5% among general population and it becomes imperative when one among every five individuals is found to be deficient [40].

There are many strategies that influence the optimum levels of 25(OH)D in general population, viz. promoting healthy lifestyle, enhancing dietary intake of naturally occurring vitamin D, adequate sun exposure, weight loss, fortification of food, and supplementation [40]. Exposure to sunlight may have other side effects, and suggesting people to eat fish may not be well appreciated by all [40]. Research has shown that vitamin D supplementation is an effective strategy in enhancing the serum 25(OH)D concentration among individuals, especially high-risk groups; however, supplementation is not an ideal public health strategy due to poor reach to low socioeconomic strata. Food fortification is one strategy, which is used by many countries (e.g., Canada, USA, Finland, and India) to target the issue of vitamin D in general population [40, 42]. Food fortification is a cost-effective and well-accepted strategy [40], but there is not enough evidence to suggest that it is beneficial in improving nutrition-related outcomes in reproductive age women.

Different guidelines recommend a supplementation of 200–800 IU/day during pregnancy and lactation, and 100–800 IU/day among adulthood except for Endocrine Society, which recommends 600–2000 IU/day [41]. However, our overview of SRs identified that the included RCTs administered higher dose of vitamin D.

There is inconclusive evidence for the effect of supplementation with calcium on bone mineral density and maternal anemia identified from our evidence synthesis. There is insufficient evidence to conclude the effect of supplementation with calcium on body weight among athletes but beneficial among overweight and obese women, and there was no effect of calcium supplementation on body weight of pregnant women. Calcium has been claimed to reduce the fat and weight of the body by various biochemical pathways, e.g., calcium stimulates the lipolysis and inhibits lipogenesis and calcium may decrease calcitriol levels by reducing adipocyte capacity to store lipids [32]. According to the WHO, daily calcium supplementation of 1.5–2.0 g oral elemental calcium is recommended for pregnant women especially from low dietary calcium intake population, to reduce the risk of pre-eclampsia [43]. According to the same guidelines, calcium supplementation is associated with an increased risk of HELLP (hemolysis, elevated liver enzymes, low platelet counts) syndrome among women. Therefore, daily calcium intake by women should be closely monitored and should not exceed the upper tolerable limits. One of the overviews [44] of SRs that looked at effects of nutrition interventions during pregnancy reported that timing of initiation of calcium supplementation during pregnancy or dietary calcium intake during the pregnancy might have an impact on pre-term birth and low birth weight. Another SR [45] that has assessed dietary calcium intake during pregnancy worldwide concludes that there is less intake of dietary calcium during pregnancy in LMICs than in HICs, therefore focusing on the need of calcium supplementation during pregnancy in LMICs.

### Limitations

We included only the studies published in English language, restriction on outcomes and interventions, and some aspects are different from that of the protocol. Calcium supplementation is one of the important interventions to prevent pre-eclampsia among pregnant women with hypertension; however, we did not consider this in the current overview as we only restricted our focus on the direct nutritional outcomes. Only SRs that included RCTs as the study design were included. The scope of this review was only restricted to maternal outcomes; future research can focus on the effect of fortification and supplementation with vitamin A, vitamin D, and calcium on the child-related nutritional outcomes.

## Conclusion

The overview of systematic reviews found that there was not enough reliable evidence available on the effect of micronutrient food fortification on nutritional status of women. There is an urgent need for conducting high-quality clinical trials and impact evaluations that give evidence on the effectiveness of fortification of food with vitamin A, vitamin D, and calcium on the nutritional status of women. Vitamin A supplementation can be given to postpartum women for increasing serum retinol levels, reiterating the WHO recommendation. Recommendation for clinical practice to use vitamin D supplementation in routine antenatal care could not be established. Calcium supplementation leads to weight loss in overweight and obese individuals.

## Supplementary information

**Additional file 1.** PRISMA checklist.

**Additional file 2.** Search Strategy.

**Additional file 3.** Characteristics of included studies.

**Additional file 4.** R-AMSTAR table.

**Additional file 5.** Detailed synthesis of the data.

**Additional file 6.** References of the primary studies. This file consists of the references for the primary studies or RCTs included in the systematic reviews that were used for data synthesis.

## Data Availability

Data will be available on request from the corresponding author.

## Abbreviations

BMD: Bone mineral density
BMI: Body mass index
CIC: Conjunctival impression cytology
CTX: Carboxy-terminal collagen crosslinks
FAO: Food and Agricultural Organization
GRADE: Grading of Recommendations Assessments Developments and Evaluations
Hb: Hemoglobin
HICs: Higher-income countries
HIV: Human immunodeficiency virus
IU: International unit
LMICs: Lower- and middle-income countries
PICO: Population Intervention Comparison Outcome
P1NP: Total procollagen type 1 N-terminal propeptide
R-AMSTAR: Revised Assessment of Multiple Systematic Reviews
RCTs: Randomized controlled trials
ROB: Risk of bias
SRs: Systematic reviews
VAD: Vitamin A deficient
WHO: World Health Organization
25(OH)D: 25-Hydroxy vitamin D
