# Supplementary material for: Effect of vitamin A, calcium and vitamin D fortification and supplementation on nutritional status of women: an overview of systematic reviews
Source: Syst Rev. 2020 Oct 27;9:248. doi: 10.1186/s13643-020-01501-8 (PMC7592561; doi:10.1186/s13643-020-01501-8)
Supplement: Supplementary file 2 — Additional file 2. Search Strategy. [file 13643_2020_1501_MOESM2_ESM.docx]

**Additional file 2**: **Search Strategy**

1. **PubMed**

Search ((((((("systematic review"[Title/Abstract] OR "Systematic Review"[Title/Abstract] OR "meta analysis"[Title/Abstract] OR "meta-analysis"[Title/Abstract] OR "Meta Analysis"[Title/Abstract] OR "Meta-analysis"[Title/Abstract]))) AND ( "0001/01/01"[PDat] : "2018/03/31"[PDat] ) AND English[lang])) AND (((((("Vitamin D" OR cholecalciferol OR ergocalciferol OR calciferol OR "vtamin D2" OR "vitamin D3" OR "hydroxyvitamin D" OR "vitamin-d" OR "vitamin-d2" OR "vitamin-d3" OR "25 hydroxyvitamin D" OR "25 hydroxyvitamin D2" OR "25 hydroxyvitamin D3" OR "25-hydroxyvitamin D" OR "25-hydroxy-vitamin D" OR "25-hydroxy-vitamin D2" OR "25-hydroxy-vitamin D3" OR "25 OHD" OR "25-OH-vitamin D" OR "25- OHD" OR calcidiol OR calcifediol OR apocal OR argonite)) OR (Folate OR "Folic Acid" OR "folic acid" OR Folacin OR "Vitamin B 9" OR "Pteroylmonoglutamic" OR "Pteroylglutamic acid")) OR ("Calcium carbonate" OR "Calcium gluconate" OR "Calcium acetate" OR "Calcium lactate" OR "Calcium citrate" OR Calcium OR Biocal OR Caltrate)) OR ("Beta carotene" OR retinol OR Retinol OR "Vitain A" OR retinoid OR Retinoid OR carotenoid OR "retinoyl palmitate")) OR (Dextran OR Venofer OR Ferric OR Ferrous OR Ferrlecit OR iron OR Ferritin OR Hematocrit OR Transferrin))) AND (fortification OR fortified OR fortify OR fortificant OR supplements OR supplement OR supplementation OR "micronutrient powder" OR enhance OR enrich)) AND (women OR woman OR females OR female OR girl OR girls) Filters: Publication date to 2018/03/31; English

**Result: 534** citations

**2. Web of Science**

| **No.** | **Search terms** | **Records** |
| --- | --- | --- |
| #1 | TOPIC: ("Systematic Review") OR TOPIC: ("systematic review") OR TOPIC: ("Meta Analysis") OR TOPIC: ("meta analysis") OR TOPIC: ("Meta-Analysis") OR TOPIC: ("meta-analysis")  Indexes=SCI-EXPANDED, SSCI, A&HCI, CPCI-S, CPCI-SSH Timespan=All years | 194,653 |
| #2 | TOPIC: (women) OR TOPIC: (woman) OR TOPIC: (girl) OR TOPIC: (girls) OR TOPIC: (female) OR TOPIC: (females)  Indexes=SCI-EXPANDED, SSCI, A&HCI, CPCI-S, CPCI-SSH Timespan=All years | 1,889,796 |
| #3 | TOPIC: (fortification) OR TOPIC: (fortificant) OR TOPIC: (fortified) OR TOPIC: (fortify) OR TOPIC: (supplementation) OR TOPIC: (supplement) ORTOPIC: (Supplement) OR TOPIC: (Supplementation) OR TOPIC: (enhance) OR TOPIC: (enrich) OR TOPIC: ("micronutrient powder")  Indexes=SCI-EXPANDED, SSCI, A&HCI, CPCI-S, CPCI-SSH Timespan=All years | 2,363,349 |
| #4 | TOPIC: ("Vitamin D") OR TOPIC: ("folic acid") OR TOPIC: (cholecalciferol) OR TOPIC: (ergocalciferol) OR TOPIC: (calciferol) OR TOPIC: ("vtamin D2") ORTOPIC: ("vitamin D3") OR TOPIC: ("hydroxyvitamin D") OR TOPIC: ("vitamin-d") OR TOPIC: ("vitamin-d2") OR TOPIC: ("vitamin-d3") OR TOPIC: ("25 hydroxyvitamin D") OR TOPIC: ("25 hydroxyvitamin D2") OR TOPIC: ("25 hydroxyvitamin D3") OR TOPIC: ("25-hydroxyvitamin D") OR TOPIC: ("25-hydroxy-vitamin D") OR TOPIC: ("25-hydroxy-vitamin D2") OR TOPIC: ("25-hydroxy-vitamin D3") OR TOPIC: ("25 OHD") OR TOPIC: ("25-OH-vitamin D") OR TOPIC: ("25- OHD") OR TOPIC: (calcidiol) OR TOPIC: (calcifediol) OR TOPIC: (apocal) OR TOPIC: (argonite)  Indexes=SCI-EXPANDED, SSCI, A&HCI, CPCI-S, CPCI-SSH Timespan=All years | 91,848 |
| #5 | TOPIC: (folate) OR TOPIC: ("Folic Acid") OR TOPIC: (folacin) OR TOPIC: ("Vitamin B 9") OR TOPIC: ("Pteroylmonoglutamic acid") OR TOPIC: ("Calcium carbonate") OR TOPIC: ("Calcium gluconate") OR TOPIC: ("Pteroylglutamic acid") OR TOPIC: ("Calcium acetate") OR TOPIC: ("Calcium lactate") ORTOPIC: ("Calcium citrate") OR TOPIC: (calcium) OR TOPIC: (biocal) OR TOPIC: (caltrate) OR TOPIC: ("Beta carotene") OR TOPIC: (retinol) OR TOPIC:("Vitamin A") OR TOPIC: (retinoid) OR TOPIC: (carotenoid) OR TOPIC: ("retinoyl palmitate") OR TOPIC: (dextran) OR TOPIC: (venofer) OR TOPIC:(ferric) OR TOPIC: (ferrous) OR TOPIC: (ferrlecit)  Indexes=SCI-EXPANDED, SSCI, A&HCI, CPCI-S, CPCI-SSH Timespan=All years | 694,065 |
| #6 | TOPIC: (iron) OR TOPIC: (ferritin) OR TOPIC: (hematocrit) OR TOPIC: (transferrin)  Indexes=SCI-EXPANDED, SSCI, A&HCI, CPCI-S, CPCI-SSH Timespan=All years | 493,768 |
| #7 | #6 OR #5 OR #4  Indexes=SCI-EXPANDED, SSCI, A&HCI, CPCI-S, CPCI-SSH Timespan=All years | 1,183,075 |
| #8 | #7 AND #3 AND #2AND #1  Refined by: DOCUMENT TYPES: (REVIEW)  Indexes=SCI-EXPANDED, SSCI, A&HCI, CPCI-S, CPCI-SSH Timespan=All years | **322** |

**3. Cochrane Library**

| **No.** | **Search terms** | **Records** |
| --- | --- | --- |
| #1 | "systematic review":ti,ab,kw or "Systematic Review":ti,ab,kw or "meta analysis":ti,ab,kw or "meta-analysis":ti,ab,kw or "Meta Analysis":ti,ab,kw (Word variations have been searched) | 48,220 |
| #2 | women or woman or females or female or girls or girl | 607,958 |
| #3 | "Vitamin D" or cholecalciferol or ergocalciferol or calciferol or "vitamin D2" or "vitamin D3" or "hydroxyvitamin D" or "vitamin-d" or "vitamin-d2" or "vitamin-d3" or "25 hydroxyvitamin D" or "25 hydroxyvitamin D2" or "25 hydroxyvitamin D3" or "25-hydroxyvitamin D" or "25-hydroxy-vitamin D" or "25-hydroxy-vitamin D2" or "25-hydroxy-vitamin D3" or "25 OHD" or "25-OH-vitamin D" or "25- OHD" or calcidiol or calcifediol or apocal or argonite or Folate or "Folic Acid" or "folic acid" or Folacin or "Vitamin B 9" or "Pteroylmonoglutamic acid" or "Pteroylglutamic acid" or "Calcium carbonate" or "Calcium gluconate" or "Calcium acetate" or "Calcium lactate" or "Calcium citrate" or Calcium or Biocal or Caltrate or "Beta carotene" or retinol or Retinol or "Vitamin A" or retinoid or Retinoid or carotenoid or "retinoyl palmitate" or Dextran or Venofer or Ferric or Ferrous or Ferrlecit or iron or Ferritin or Hematocrit or Transferrin or "micronutrient powder" | 47,075 |
| #4 | fortification or fortified or fortify or supplementation or supplements or enrich or enhance or supplement | 79,735 |
| #5 | #1 and #2 and #3 and #4 | **561** |

**4. Scopus**

( ( TITLE-ABS-KEY ( fortification )  OR TITLE-ABS-KEY ( fortificant )  OR TITLE-ABS-KEY ( fortified )  OR TITLE-ABS-KEY ( fortify )  OR TITLE-ABS-KEY ( supplements )  OR TITLE-ABS-KEY ( supplementation )  OR TITLE-ABS-KEY ( enhance )  OR TITLE-ABS-KEY ( enrich )  OR TITLE-ABS-KEY ( supplement ) ) )  AND ( ( TITLE-ABS-KEY ( "Systematic Review" )  OR TITLE-ABS-KEY ( "systematic review" )  OR TITLE-ABS-KEY ( "meta analysis" )  OR TITLE-ABS-KEY ( "meta-analysis" )  OR TITLE-ABS-KEY ( "Meta-Analysis" )  OR TITLE-ABS-KEY ( "Meta Analysis" ) ) )  AND ( ( TITLE-ABS-KEY ( women )  OR TITLE-ABS-KEY ( woman )  OR TITLE-ABS-KEY ( female )  OR TITLE-ABS-KEY ( females )  OR TITLE-ABS-KEY ( girl )  OR TITLE-ABS-KEY ( girls ) ) )  AND ( ( TITLE-ABS-KEY ( dextran )  OR TITLE-ABS-KEY ( venofer )  OR TITLE-ABS-KEY ( ferric )  OR TITLE-ABS-KEY ( ferrous )  OR TITLE-ABS-KEY ( ferrlecit )  OR TITLE-ABS-KEY ( ferritin )  OR TITLE-ABS-KEY ( hematocrit )  OR TITLE-ABS-KEY ( transferrin )  OR TITLE-ABS-KEY ( "Beta carotene" )  OR TITLE-ABS-KEY ( retinol )  OR TITLE-ABS-KEY ( retinol )  OR TITLE-ABS-KEY ( retinoid )  OR TITLE-ABS-KEY ( retinoid )  OR TITLE-ABS-KEY ( carotenoid )  OR TITLE-ABS-KEY ( "retinoyl palmitate" )  OR TITLE-ABS-KEY ( "Calcium carbonate" )  OR TITLE-ABS-KEY ( "Calcium gluconate" )  OR TITLE-ABS-KEY ( "Calcium acetate" )  OR TITLE-ABS-KEY ( "Calcium lactate" )  OR TITLE-ABS-KEY ( "Calcium citrate" )  OR TITLE-ABS-KEY ( calcium )  OR TITLE-ABS-KEY ( iron )  OR TITLE-ABS-KEY ( "Vitamin A" )  OR TITLE-ABS-KEY ( biocal )  OR TITLE-ABS-KEY ( calcitate )  OR TITLE-ABS-KEY ( caltrate )  OR TITLE-ABS-KEY ( folate )  OR TITLE-ABS-KEY ( "Folic Acid" )  OR TITLE-ABS-KEY ( "folic acid" )  OR TITLE-ABS-KEY ( folacin )  OR TITLE-ABS-KEY ( " Vitamin B 9" )  OR TITLE-ABS-KEY ( " Pteroylmonoglutamic acid" )  OR TITLE-ABS-KEY ( " Pteroylglutamic acid" )  OR TITLE-ABS-KEY ( "Vitamin D" )  OR TITLE-ABS-KEY ( cholecalciferol )  OR TITLE-ABS-KEY ( ergocalciferol )  OR TITLE-ABS-KEY ( calciferol )  OR TITLE-ABS-KEY ( "vitamin D2" )  OR TITLE-ABS-KEY ( "vitamin D3" )  OR TITLE-ABS-KEY ( "hydroxyvitamin D" )  OR TITLE-ABS-KEY ( "vitamin-d " )  OR TITLE-ABS-KEY ( "vitamin-d2 " )  OR TITLE-ABS-KEY ( "vitamin-d3 " )  OR TITLE-ABS-KEY ( "25 hydroxyvitamin D" )  OR TITLE-ABS-KEY ( "25 hydroxyvitamin D2" )  OR TITLE-ABS-KEY ( "25 hydroxyvitamin D3" )  OR TITLE-ABS-KEY ( "25-hydroxyvitamin D " )  OR TITLE-ABS-KEY ( "25-hydroxy-vitamin D" )  OR TITLE-ABS-KEY ( "25-hydroxy-vitamin D2" )  OR TITLE-ABS-KEY ( "25-hydroxy-vitamin D3" )  OR TITLE-ABS-KEY ( "25OHD " )  OR TITLE-ABS-KEY ( "25-OH-vitamin D" )  OR TITLE-ABS-KEY ( "25- OHD" )  OR TITLE-ABS-KEY ( calcidiol )  OR TITLE-ABS-KEY ( calcifediol )  OR TITLE-ABS-KEY ( apocal )  OR TITLE-ABS-KEY ( argonite ) ) )  AND ( LIMIT-TO ( SUBJAREA ,  "MEDI" )  OR LIMIT-TO ( SUBJAREA ,  "NURS" )  OR LIMIT-TO ( SUBJAREA ,  "AGRI" )  OR LIMIT-TO ( SUBJAREA ,  "SOCI" )  OR LIMIT-TO ( SUBJAREA ,  "Undefined" ) )  AND ( LIMIT-TO ( DOCTYPE ,  "re" ) )  AND ( LIMIT-TO ( LANGUAGE ,  "English" ) )

**Results: 605** citations
