# Supplementary material for: Effect of vitamin A, calcium and vitamin D fortification and supplementation on nutritional status of women: an overview of systematic reviews
Source: Syst Rev. 2020 Oct 27;9:248. doi: 10.1186/s13643-020-01501-8 (PMC7592561; doi:10.1186/s13643-020-01501-8)
Supplement: Supplementary file 3 — Additional file 3. Characteristics of included studies. [file 13643_2020_1501_MOESM3_ESM.docx]

**Additional file 3: Table of Characteristics of included systematic reviews***

| **Author ID & methodological quality of SR** | **Population, Study design and Region** | **Intervention** | **Outcomes** | **Author’s conclusion** |
| --- | --- | --- | --- | --- |
| Arthur et al., 2015  Low | **Participants:** Women, children  **Study design:** Observational and interventional  Total 67 studies included  **Geographical Location:**  Asia, Africa and Pacific region | Micronutrient fortification and supplementation like vitamin A, zinc, calcium  Malnutrition prevention campaigns  Nutrition programmes | Prevalence of malnutrition  Determinants of malnutrition  Effects of malnutrition  Interventions needed to tackle malnutrition including supplementation and fortification  Outcomes related to micronutrient supplementation and fortification like: increase in maternal vitamin A liver reserves, risk of anaemia, postpartum weight loss of mothers, bone mineral density of women | Exposure to diseases like HIV and malaria are important biological determinants of malnutrition  Wealth, education and paternal involvement in caring for children were important social factors affecting malnutrition  Mortality, morbidity due to diseases like pneumonia, malaria, poor development for e.g. stunting and low cognitive ability till five years of age are some of the effects of malnutrition  Breastfeeding and administration of fortified food and supplementation with vitamin A, zinc, calcium, and multiple micronutrients are important interventions for tacking malnutrition. Supplementation and fortification with these micronutrients have resulted in positive maternal and infant outcomes. |
| Buppasiri et al., 2015  High | **Participants:** Pregnant women  **Geographical location:** Argentina, Australia, Columbia, Egypt, Ecuador, Gambia, Guatemala, Hong Kong, India, Iran, Mexico, South Africa, United States and Vietnam | Calcium (calcium carbonate, calcium gluconate, calcium lactate and combined calcium) supplementation during pregnancy | **Primary outcomes:** 1) Maternal outcomes: Preterm birth less than 37 weeks’ gestation 2) Infant outcomes: Low birth weight (less than 2500 g).  **Secondary outcomes:** 1) Maternal outcomes: Preterm birth less than 34 weeks’ gestation, Maternal weight gain, Maternal bone mineral density (BMD) measured by dual- energy x-ray absorptiometry (osteopenia is classified as BMD between -1 and -2.5 SD; osteoporosis is classified as BMD less than -2.5 SD), Leg cramps, Backache, Tetany, Incidence of fracture, Duration of breastfeeding, Tremor, Paraesthesia, Mother admitted to an intensive care unit, Maternal death, Mode of birth (vaginal birth, Instrumental vaginal birth, caesarean section), Postpartum haemorrhage.  2) Foetal and neonatal outcomes: Stillbirth or foetal death (foetus died in uterus after 20 weeks’ gestation or during labour and delivery), Neonatal death (baby died in first 28 days of life), Perinatal mortality (stillbirth and neonatal death), Admission to neonatal intensive care unit, Birthweight, Birth length, Head circumference, Intrauterine growth restriction, Neonatal BMD (measured by single-photon absorptiometry or dual-energy x-ray absorptiometry), Osteopenia, Rickets, Fracture. 3) Adverse outcomes: Side effects of calcium supplementation, Compliance, Satisfaction, Urinary stones, Urinary tract infection, Nephrocalcinosis, Impaired renal function, Maternal anaemia. | No reduction in preterm birth (less than 37 weeks).  No evidence for pregnancy outcomes other than pregnancy induced hypertension.  There are few short term benefits of calcium supplementation like foetal birth weight and neonatal bone mineral density |
| Caminha, et al., 2009  Low | **Participants-**Post-partum women  **Study Design-**  14 RCTs including 39,954 participants  **Geographical location-**  India, Brazil, Peru, Ghana and Bangladesh | Vitamin A supplementation  Dose: 200,000 IU given in a range of 12 hrs. To 42 days after delivery in seven RCTs  300,000 IU given 7-21 days after delivery 400,000 v/s 200,000 IU within 6 weeks after delivery, in three RCTs 400,000 vs placebo  Given in from 24 hrs-96 hrs. after delivery in three RCTs | Maternal breast milk retinol levels and serum retinol levels of the women | **Retinol levels in milk:** Nine out of eleven studies reported that retinol levels had increased, in comparison with the baseline or when compared to control.  **Serum retinol responses:** elevation in the serum retinol content was noticed in four out of nine studies. |
| Chakhtoura et al., 2017  Moderate | **Participants and region**: Healthy infants, children, adolescents or pregnant women, from the MENA^[[1]](#footnote-1)^ region  **Study design**: RCTs: 10 studies in pregnant women, 4 in children and adolescents and one study in infants. | Vitamin D2 or D3, of any dose given at least once monthly and at least for a 3-month duration.  Exclusion: studies conducted in children with rickets, in  Institutionalized/hospitalized individuals, those with chronic illnesses (kidney, liver or heart failure), and in the presence of conditions or drug therapy that affect vitamin D metabolism (malabsorption, anticonvulsants, steroids, anti-fungal medications). Studies administering vitamin D supplementation as a fortified food or in the active form. | 1. 25 (OH)D level (for all groups). 2. PTH level and serum calcium (Children and adolescents) 3. serum calcium, glycaemic indices (fasting blood glucose, HOMA-IR, insulin levels), lipid profiles (HDL level) and inflammatory markers (hs - CRP, diastolic BP) [Pregnant women] | In children, adolescents and pregnant women from the MENA, an intermediate vitamin D dose of 1,000–2,000 IU daily seems necessary to allow for the majority of the population to reach the desirable 25(OH)D level of 20 ng/ml, as recommended by the National Academy of Medicine, Institute of Medicine. Data on the skeletal and extra-skeletal outcomes and on the long term safety of high vitamin D doses in our region are scarce. |
| Christesen et al., 2012  Moderate | **Participants**: n=857, pregnant women.  **Region**: 1 study: Asian in UK, 1 ethnic groups in UK, 1 Hispanic, white black women in US, 1 Indian, 2 France.  **Study design**: 7 RCTs | Vitamin D- 2*600,000 IU 2 times (1 study)  Vitamin D3/2- 1000 IU/day (2 studies), 1000 IU/day in one arm and 200,000 IU single dose (1 study), 120,000 IU, 2 times in pregnancy (1 study), 200,000 IU single dose in one arm and 60,000 IU single dose in another arm(1 study), 4000 IU/day in one arm and 2000 IU/day in another arm. | Pregnancy related outcomes 25OHD levels; weight, length of gestation, mode of delivery, fertility, preeclampsia, gestational diabetes | Increase in 25 OHD level at delivery compared with lower doses or no supplementation  Backache, leg pain, general weakness and cramps were much more common in un-supplemented group |
| Cumming, 1990  Low | **Participants:** Pre-menopausal and post-menopausal women  **Study Design:**  Fourty nine included studies.  Out of these 13 RCTs and rest observational studies  **Geographical Location:** Not Reported | Ca supplementations only; not in combination with vitamin D (more than 400 IU), oestrogen or exercise. | Measure of bone mass: Percentage of bone loss or the actual rate at which bone was lost (in grams per centimeter-cm2 or cm3). | -Mean effect of calcium: 0.02 % difference per year (percent bone loss per year in the group given calcium supplements minus percent bone loss per year in control group)  -In premenopausal women, only left humerus BMC loss was significantly reduced by Ca supplementation  -Oral calcium supplementation had a positive effect on post- menopausal women at all the bone sites except the vertebrae |
| Das et al., 2013  Moderate | **Participants:** Children and adolescents of all age group (2-18 yrs.)  Pre-pregnant women, reproductive aged women (18-49 yrs) and post- menopausal women  **Study Design:** 201 studies including 125 RCTs, 7 Quasi experimental studies, and 69 before after studies  **Geographical location:**  Global | **For Children**: Food fortified with iron, zinc, vitamin A, D, calcium, folic acid, iodine and multiple micronutrients  Sodium iron ethyl-enediaminetetraacetate (NaFeEDTA), ferrous sulphate, ferrous pyrophosphate, unspecified elemental iron and hydrogen reduced iron, zinc sulphate, zinc chloride, zinc oxide, zinc acetate, monosodium glutamate, sugar, flour  **For women:** Iron, folate, iodine, multiple micronutrients, vitamin D and Calcium fortification was assessed  The choice of food vehicle varied for both children and women | **Iron:** Serum ferritin, serum transferrin, haemoglobin and anaemia  **Iodine:** Serum thyroxin levels, prevalence of hypothyroidism and urinary iodine concentration  **Vitamin A:** Serum vitamin A concentration, vitamin A deficiency and haemoglobin  **Vitamin D/Calcium:** Serum 25-hydroxy vitamin D3 concentration, alkaline phosphatase and serum parathyroid hormone  **Zinc:** Serum zinc concentration, height velocity, alkaline phosphatase, haemoglobin and serum copper concentration  **Folate:** Serum folate concentration, red blood cell folate concentration, folate deficiency, twinning, incidence of neural tube defects, anencephaly and spina bifida  **Multiple micronutrients:**  Serum ferritin, serum zinc, serum retinol, vitamin A deficiency, iron deficiency anaemia, haemoglobin levels, height for age Z-scores, weight for age Z-scores, weight for height Z-scores and morbidity | Fortification improved outcomes among children and women but authors recommended that more research is needed. |
| McCauley, et al., 2015  High | **Population-**  Pregnant women receiving vitamin A supplementation  **Study Design**- 19 RCTS consisting of 310,000 women. Three trials were cluster randomized and two were quasi-experimental studies.  **Geographical Region-**  Indonesia, USA, UK, Nepal, South Africa,  Tanzania, Ghana and Bangladesh. | Vitamin A (or one of its derivatives) supplementation, alone or in combination with other supplements compared with a control group. | **Primary Outcomes:** Maternal mortality, perinatal mortality  **Secondary outcomes**: Neonatal mortality, stillbirths, maternal anaemia (Hb less than 11 gm/dl), maternal clinical infection, maternal night blindness, preterm birth (less than 37 weeks gestation), neonatal anaemia, neonatal clinical infection, congenital malformations, low birthweight (less than 2.5 kg) | No evidence for role of antenatal vitamin A supplementation in reducing maternal or infant mortality  Antenatal vitamin A supplementation along with iron and folic acid reduces the risk of maternal anaemia for VAD and HIV positive women  Reduction in maternal infection was reported when vitamin A was given in antenatal period or around the time of delivery |
| Neves, et al., 2015  Moderate | **Participants-**Adult healthy women  **Study Design**- 7 RCTs with 925 participants  **Geographical Location-**  Brazil | Vitamin A in the form of retinol palmitate  Dose: 200,000 IU; 400,000 IU  Given either immediate postpartum or from 16 hrs. to 8/10 days after delivery to postpartum women | Retinol and immunoglobulin content of the maternal milk,  Mother-child binomial vitamin A status | **Retinol levels in colostrum:** One study reported no significant difference in the retinol levels of the colostrum, when supplemented with double dose of 200,000 IU vitamin A and control. Another study reported a significant difference in retinol content of the colostrum, after 24 hours of supplementation, when supplemented with 200,000 IU 16 hours after delivery.  An increase in sIgA contents of the colostrum after supplementation of 200,000 IU vitamin A was reported by one study.  **Retinol levels in breast milk:** Three studies reported a significant difference in retinol contents of breast milk for group supplemented with 200,000 IU vitamin A and control. |
| Oliveira, et al., 2016  High | **Participants-**Postpartum women from vitamin A deficiency areas  **Study Design-**  14 RCTs with 25,758 women and infant pairs  **Geographical Location-**  India, Bangladesh, Indonesia, Tanzania, Gambia, Zimbabwe, Kenya, Ghana, Peru and Brazil | Maternal vitamin A supplementation (beta-carotene or retinyl palmitate in oil or water-miscible formulation) alone or in combination with other micronutrients (examples: iron, folic acid, vitamin E) commenced at any time during the postpartum period, that is, within 24 hours after birth until the sixth week postpartum were eligible. | **Primary Outcomes**  Maternal   1. Mortality. 2. Morbidity (febrile illness, respiratory tract infection, diarrhoea, anaemia and others). 3. Adverse effects of vitamin A within three days after receiving supplement.   Infant   1. Mortality. 2. Morbidity (febrile illness, respiratory tract infection, diarrhoea, anaemia and others). 3. Adverse effects of vitamin A supplementation within three days after receiving supplement.   **Secondary Outcomes**   1. Serum retinol concentration. 2. Vitamin A hepatic reserves (MRDR or RDR). 3. Breast milk retinol concentration. 4. VAD (clinical: impaired visual adaptation to darkness, night blindness, xerophthalmia; and subclinical: abnormal conjunctival impression cytology (CIC))   Infant   1. Serum retinol concentration. 2. Vitamin A hepatic reserves (relative dose response (RDR) or modiﬁed relative dose response (MRDR)).   Clinical VAD (clinical: signs of xerophthalmia). | Vitamin A supplementation given to postpartum women residing in vitamin A deficiency regions, increases the serum retinol and breast milk retinol concentration  No significant differences observed for maternal and infant mortality and morbidity in between vitamin A supplementation and control  No adverse effects of vitamin A supplementation reported for mothers or infants |
| Onakpoya, et al., 2011  Moderate | **Participants:** overweight and obese females  **Study Design:** 7 RCTs with 794 participants  **Geographical Location:**  Global | calcium supplementation | Body Weight, Body Fat, BMI(Body Mass Index) | Calcium supplementation for six months results in significant weight loss of obese and overweight individuals |
| Reid, Bolland & Grey, 2014  Moderate | **Participants**: n=4082, 92% women, in six studies average age was younger than 50. (unclear minimum and maximum age).  **Region and ethnicity**: 19 studies included mainly white populations, two were done in African-American, one among Pakistani immigrants in Denmark, one study Bangladesh. Two studies included overweight population.  **Study design**: RCTs (n=23) comparing intervention that differed only in vitamin D content. 18 RCTs (One study used a crossover design and rest parallel) were placebo controlled, two had open control arm, three were comparison between two different doses of vitamin D | Vitamin D3 or D2 but not a vitamin D metabolite. If co-interventions were given (e.g. calcium), they had to be equally distributed in all groups.  A wide range of baseline concentrations of 25-hydroxyvitamin D were reported. The mean level was less than 30 nmol/L in five studies, 30–50 nmol/L in three studies, 50–75 nmol/L in 11 studies, and more than  75 nmol/L in one study (healthy Australian women in early post-menopause). In 12 studies, calcium supplements were given to all trial groups. Two studies had average total calcium intakes of less than  750 mg per day.  Three small studies were of 6 months duration, eight for 1 year, and 12 for 2–5 years. The weighted mean trial duration was 23·5 months.  Various supplement regimens were assessed. Most trials used daily oral dosing, although in two studies, supplementation was given only for nine months of each year. Four studies dosed participants at weekly or monthly intervals, and two studies gave annual intramuscular injections of 300 000 units. When doses  are averaged, 500 IU per day or less was given in six studies, 500–799 IU per day in four studies, and 800 IU per day or more in 13 studies. Three studies had three groups (two different vitamin D doses and a control group) | Bone mineral density (% change) (or in the case of forearm assessment, bone mineral content) had to be available, irrespective of whether this was the primary endpoint. BMD was measured at one to five sites (lumbar spine, femoral neck, total hip, trochanter, total body, or forearm) in each study. | The evidence is scarce on benefit of vitamin D supplementation on bone density targeting of low-dose vitamin D supplements only to individuals who are likely to be deficient, will be beneficial. |
| Roth et al., 2017  High | **Participants**: 5706 pregnant women, pregnant at the time of enrolment or enrolled before pregnancy and then followed-up in pregnancy  **Region**: Global  **Study design**: RCTs. 43 eligible trials had 55 intervention-control arm comparisons | Supplementation with prenatal vitamin D. Supplementation was vitamin D alone or in combination with a co-intervention that was similar across multiple arms. Supplementation could be either Vitamin D2 or D3, administered as any dose and by any route (oral or intramuscular), at any frequency i.e. ‘regular dosing’ in which supplementation was offered at least three times in a regular/recurrent manner (e.g. daily, monthly) and ‘bolus’ dose regimens in which the supplement was administered only once or twice.  At least one trial group received placebo, no vitamin D or up to 600 IU/day (or less frequent dose e.g. 4200 IU/week) during pregnancy. | Maternal Outcomes: 1. Pre-eclampsia 2. Gestational Diabetes 3. Intrauterine death/stillbirth 4. Caesarean section 5. Preterm labour 6. Maternal biochemical adverse events (hypercalcemia, hypercalciuria, hypocalcaemia) 7. Admission to hospitals 8. Serum 25(OH) concentration at/near delivery. | The included RCTs were of low quality, small and were not intended to measure clinical outcomes of the interest. Pooling of data was possible but heterogeneity restricted the interpretations. |
| Trowman et al., 2006  Moderate | **Participants:** Non pregnant, non-lactating adults above 18 yrs. Of age  **Study Design:** 11 trials with 1127 participants  **Geographical Location:**  UK, USA, Asia | Calcium fortified products, supplementation, or increased provision of dairy products. Calcium level of 300 mg per day or more were included. | Body Weight | No evidence of changes in body weight after calcium supplementation or consumption of increased dairy products |
| Thorne—Lyman & Fawzi, 2012  Moderate | **Participants-** Pregnant women  Three out of seventeen studies included HIV positive participants  **Study Design-**  17 RCTs  Three large cluster randomized trials.  **Geographical Location-**  India, South Africa, Ghana, Indonesia, Tanzania, Malawi, China, Nepal, Bangladesh | Vitamin A supplementation  Dose varied from 3333-10,000 IU per day  Two studies in HIV positive women included two intervention arms i.e. (1) 5000 IU vitamin A per day and 30 mg beta carotene (2) 200,000 IU dose of vitamin A at delivery | **Primary and sub-outcomes**  1. Low birthweight due to intra-uterine growth restriction  1a. Small-for-gestational age  1b. Low birthweight  1c. Mean birthweight  1d. Weight gain during pregnancy  1e. Average heel-crown length at delivery  2. Preterm birth  2a. Preterm birth  2b. Early preterm birth  2c. Mean gestational age at delivery  3. Neonatal growth, morbidity and mortality  3a. Early infant mortality (first 6 weeks)  4. Infant and child growth, morbidity and mortality  4a. Mean weight  4b. Mean height  4c. Low weight-for-age  4d. Low height-for-age  4e. All-cause mortality  4f. Mortality due to measles, diarrhoea, acute lower  respiratory infection or malaria  4g. Mother-to-child transmission of HIV  4h. Morbidity (diarrhoea, measles, malaria, acute lower  respiratory infection)  4i. Child anaemia and mean haemoglobin  5. Maternal mortality and pregnancy complications  5a. Mortality (all cause)  5b. Mortality due to haemorrhage, sepsis or obstructed labour  5c. Hospital admissions from complications or Caesarean section  5d. Pre-eclampsia  6. Maternal nutritional status  6a. Anaemia and mean haemoglobin | No effect on maternal or infant mortality  Vitamin A supplementation reduces the risk of maternal anaemia and increases the maternal haemoglobin levels during pregnancy, especially during co intervention with iron and folate.  Vitamin A supplementation reduces the risk of low birth weight among HIV positive women.  No evidence of significant adverse effects for vitamin A supplementation was reported |
| Regil et al., 2016  High | **Participants**: Pregnant women of any gestational or chronological age, parity (number of births) and number of fetuses. Pregnant women with pre-existing conditions (i.e. gestational diabetes) were excluded. The sample size from all the studies was small and ranged between  40 and 400 women. Women were recruited and received the supplements at 20 or more weeks’ gestation  **Region**: Global. Latitude of the settings was north of the Tropic of Cancer (n=13), One trial was conducted between the Tropics of Cancer and Capricorn, and one study was conducted just were the tropic of Capricorn lies  **Study design**: RCTs and quasi-randomised trials with randomisation at either individual or cluster level, but found only RCTs (n=15) with individual randomisation. Cross-over trials or any other observational designs (e.g. cohort or case-control studies) were excluded. | Vitamin D supplementation during pregnancy irrespective of dose, duration or time of commencement of supplementation Included trials testing vitamin D alone or in combination with other micronutrients as long as the intervention and the control group were treated similarly. Nine trials compared provision of oral vitamin D supplement in comparison with placebo or no intervention, while six trials compared provision of oral vitamin D plus calcium supplements versus no intervention or placebo. The dose of vitamin D provided varied. The daily doses used were 200 IU vitamin D in five trials, 400 IU vitamin D in two trials, 800 IU vitamin D in another trial, 1000 IU vitamin D in four trials, 1200 IU vitamin D in one trial and 2000 IU vitamin D in a one group in one trial.  For single-dose supplementation of vitamin D, the dose varied from 200,000 IU vitamin D in a group in one study, 600,000 IU vitamin D in one trial, and 35,000 IU vitamin D per week. In one study dose varied from one dose of  60, 000 IU (if serum 25(OH)-D levels were > 50 nmol/L), two doses of 120,000 IU (if serum 25(OH)-D levels were 25-50 nmol/L), or four doses of 120,000 IU (if serum 25(OH)-D levels < 25 nmol/L).  The vitamin D was provided in the form of cholecalciferol-D3 in 10 trials and as ergocalciferol-D2 in three trials. Two trials do not report the vitamin D form used.  The doses of calcium provided along with the vitamin D ranged from 375 mg of calcium (one study), 500 mg of calcium as calcium carbonate (two studies), 600 mg  Calcium (two studies) and 1250 mg of calcium as calcium carbonate (one study).  In nine trials there was a group receiving the vitamin D supplements daily. In three trials the single dose provided was either once weekly or once a month. In one study, the supplementation was either once at 20 weeks, twice at 20 and 24 weeks or four times at 20, 24, 28 and 32 weeks of gestation. | **Primary outcomes**: Maternal- Pre-eclampsia (as deﬁned by trialists). Gestational diabetes (as deﬁned by trialists). Vitamin D concentration at term (25-hydroxyvitamin D in nmol/L). Adverse effects (e.g. hypercalcaemia, kidney stones). Infants: Preterm birth (less than 37 weeks’ gestation). Low birthweight (less than 2500 g).  **Other secondary maternal and infant outcomes.**  Different laboratory methods were used to measure vitamin D status as serum 25(OH)D concentrations. Three trials used a commercial  ELISA kit (Immuno Diagnostic Systems); one trial used a chemiluminescent enzyme-labelled immunometric assay; one trial used isotope-dilution liquid chromatography-tandem mass spectrometry; one used high-performance liquid chromatography tandem mass spectroscopy. Two trials used a competitive protein binding assay, and one trial used a radioligand assay. In one trial, the laboratory method was not reported. | Supplementing pregnant women with vitamin D in a single or continued dose increases serum 25-hydroxyvitamin D at term but the results were highly variable. The clinical significance of the increased serum 25-hydroxyvitamin D concentrations is unclear and results should be interpreted with caution, as only a few small trials of low quality assessed these outcomes. Also, there was heterogeneity in the results on serum 25-OH D. This variability and inconsistency could be related to the differences in methods used to assess this outcome in the included trials. The evidence on whether vitamin D supplementation should be given as a part of routine antenatal care to all women to improve maternal and infant outcomes therefore remains unclear. The clinical significance of this finding and the potential use of this intervention as a part of routine antenatal care are yet to be determined.  While, there is some indication that vitamin D supplementation could reduce the risk of pre-eclampsia and increase length and head circumference at birth, further rigorous randomised trials are required to confirm these effects. Currently, the number of high quality trials with large sample sizes and outcomes reported, including data on adverse effects, is too limited to draw definite conclusions on its usefulness and safety. |

* The list of RCTs those were included by systematic reviews is provided in Additional file 6.

1. MENA region as defined by World Bank includes: Algeria, Bahrain, Djibouti, Egypt, Jordan, Iran, Iraq, Kuwait, Lebanon, Libya, Malta, Morocco, Oman, Palestine/Israel, Qatar, Saudi Arabia, Syria, Tunisia, United Arab Emirates and Yemen [↑](#footnote-ref-1)
