# Supplementary material for: Effect of vitamin A, calcium and vitamin D fortification and supplementation on nutritional status of women: an overview of systematic reviews
Source: Syst Rev. 2020 Oct 27;9:248. doi: 10.1186/s13643-020-01501-8 (PMC7592561; doi:10.1186/s13643-020-01501-8)
Supplement: Supplementary file 5 — Additional file 5. Detailed synthesis of the data. [file 13643_2020_1501_MOESM5_ESM.docx]

| **Additional file 4: Table on R-AMSTAR score of included systematic reviews** | | | | | | | | | | | | | | | |  |
| --- | --- | --- | --- | --- | --- | --- | --- | --- | --- | --- | --- | --- | --- | --- | --- | --- |
| **Item. No#** | **Das, et al.,**  **2013** | **Roth et al.,**  **2017** | **Trowman, et al., 2006** | **Cumming, 1990** | **Reid, Bolland, & Grey, 2014** | **Chakhtoura, et al., 2017** | **Buppasiri, et al., 2015** | **Onakpoya, et al., 2011** | **Caminha, et al., 2009** | **Neves, et al.,**  **2015** | **Oliveira, et al., 2016** | **Thorne-Lyman, Fawzi, 2012** | **McCauley, et al., 2015** | **De Regil, etal., 2016** | **Christesen, et al., 2012** | **Arthur, et al., 2015** |
| **1(A)** | N | Y | N | N | U | Y | U | N | N | N | U | U | U | U | N | U |
| **1(B)** | Y | Y | Y | Y | Y | Y | Y | Y | Y | Y | Y | N | Y | Y | Y | U |
| **1(C)** | Y | Y | Y | Y | Y | Y | Y | Y | Y | U | Y | N | Y | Y | Y | NA |
| **Score** | **3** | **4** | **3** | **3** | **3** | **4** | **3** | **3** | **3** | **2** | **3** | **1** | **3** | **3** | **3** | **2** |
| **2(A)** | Y | Y | U | U | Y | Y | Y | Y | U | N | Y | N | Y | Y | Y | U |
| **2(B)** | Y | Y | U | U | Y | Y | Y | Y | U | Y | Y | N | Y | Y | Y | N |
| **2(C)** | Y | Y | Y | U | Y | Y | Y | Y | U | N | Y | U | Y | U | Y | U |
| **Score** | **4** | **4** | **2** | **1** | **4** | **4** | **4** | **4** | **1** | **2** | **4** | **1** | **4** | **3** | **4** | **1** |
| **3(A)** | Y | Y | Y | N | Y | Y | Y | Y | N | Y | Y | Y | Y | Y | Y | U |
| **3(B)** | Y | Y | Y | Y | Y | Y | Y | Y | Y | Y | Y | Y | Y | Y | Y | U |
| **3(C)** | N | Y | Y | Y | Y | U | Y | Y | Y | Y | Y | Y | Y | Y | Y | Y |
| **3(D)** | Y | Y | Y | Y | U | Y | Y | Y | N | N | Y | Y | Y | Y | N | U |
| **3(E)** | Y | U | U | Y | U | U | Y | Y | N | N | Y | Y | Y | Y | U | N |
| **Score** | **4** | **4** | **4** | **4** | **3** | **3** | **4** | **4** | **2** | **3** | **4** | **4** | **4** | **4** | **3** | **1** |
| **4(A)** | Y | Y | U | N | N | U | Y | Y | N | N | U | N | N | Y | U | N |
| **4(B)** | Y | Y | N | Y | N | Y | Y | Y | N | N | Y | N | Y | N | Y | N |
| **4(C)** | NA | U | U | U | U | U | U | U | U | U | U | U | U | NA | NA | N |
| **4(D)** | Y | Y | U | U | U | Y | Y | Y | Y | Y | Y | N | Y | Y | N | U |
| **Score** | **4** | **4** | **1** | **2** | **1** | **3** | **4** | **4** | **2** | **2** | **3** | **1** | **3** | **3** | **3** | **1** |
| **5(A)** | Y | Y | Y | Y | Y | Y | Y | N | Y | Y | Y | Y | Y | Y | Y | N |
| **5(B)** | N | U | N | N | N | N | Y | N | N | N | Y | N | Y | Y | N | N |
| **5(C)** | U | Y | Y | N | Y | Y | Y | Y | N | Y | Y | U | Y | Y | Y | N |
| **5(D)** | U | Y | N | N | N | N | Y | Y | U | Y | Y | Y | Y | Y | N | U |
| **Score** | **1** | **3** | **2** | **1** | **2** | **2** | **4** | **1** | **1** | **3** | **4** | **2** | **4** | **4** | **2** | **1** |
| **6(A)** | A | Y | Y | Y | Y | Y | Y | N | Y | Y | Y | Y | Y | Y | Y | N |
| **6(B)** | N | Y | Y | N | Y | Y | Y | N | Y | N | Y | Y | Y | Y | N | N |
| **6(C)** | N | Y | Y | N | Y | Y | Y | U | N | N | Y | Y | Y | Y | N | N |
| **Score** | **2** | **4** | **4** | **2** | **4** | **4** | **4** | **1** | **3** | **2** | **4** | **4** | **4** | **4** | **2** | **1** |
| **7(A)** | Y | Y | U | N | Y | Y | Y | Y | U | Y | Y | U | Y | Y | N | N |
| **7(B)** | Y | Y | U | N | U | U | Y | Y | NA | N | Y | U | Y | Y | U | N |
| **7(C)** | Y | U | N | N | N | N | Y | N | U | N | U | Y | Y | Y | N | N |
| **7(D)** | Y | U | N | N | N | N | Y | N | N | N | Y | Y | Y | Y | N | N |
| **Score** | **4** | **2** | **1** | **1** | **1** | **1** | **4** | **2** | **1** | **1** | **3** | **2** | **4** | **4** | **1** | **1** |
| **8(A)** | N | Y | U | N | N | Y | Y | U | NA | N | Y | Y | Y | Y | U | N |
| **8(B)** | Y | Y | N | N | N | U | Y | N | NA | N | N | Y | N | U | N | N |
| **8(C)** | Y | Y | N | Y | Y | Y | Y | Y | U | Y | Y | Y | Y | Y | U | Y |
| **8(D)** | N | Y | N | U | NA | U | U | N | U | Y | Y | U | Y | Y | U | Y |
| **Score** | **2** | **4** | **1** | **1** | **2** | **2** | **3** | **1** | **2** | **2** | **3** | **3** | **3** | **3** | **1** | **2** |
| **9(A)** | Y | Y | Y | N | N | Y | Y | Y | N | N | Y | Y | Y | Y | N | N |
| **9(B)** | Y | Y | Y | N | Y | Y | Y | Y | NA | NA | Y | Y | Y | Y | N | NA |
| **9(C)** | Y | Y | Y | N | Y | Y | Y | Y | N | N | Y | Y | Y | Y | N | N |
| **9(D)** | Y | Y | Y | N | Y | U | Y | NA | NA | NA | Y | Y | Y | Y | N | NA |
| **9(E)** | NA | NA | NA | N | U | U | NA | Y | N | N | NA | NA | NA | NA | N | NA |
| **Score** | **4** | **4** | **4** | **1** | **3** | **3** | **4** | **4** | **2** | **2** | **4** | **4** | **4** | **4** | **1** | **2** |
| **10(A)** | Y | Y | Y | N | Y | U | Y | Y | N | NA | N | N | N | N | U | N |
| **10(B)** | Y | U | Y | N | Y | N | Y | Y | NA | NA | NA | N | NA | NA | U | N |
| **10(C)** | N | U | U | N | Y | N | U | N | NA | NA | NA | N | NA | NA | U | N |
| **Score** | **3** | **2** | **3** | **1** | **4** | **1** | **3** | **3** | **3** | **4** | **3** | **1** | **4** | **3** | **1** | **1** |
| **11(A)** | N | Y | N | N | Y | Y | Y | Y | N | Y | Y | Y | Y | Y | Y | Y |
| **11(B)** | N | Y | U | U | Y | N | Y | Y | N | Y | Y | N | Y | Y | U | Y |
| **11(C)** | U | N | U | N | N | U | U | U | U | U | Y | U | U | Y | U | U |
| **Score** | **1** | **3** | **1** | **1** | **3** | **2** | **3** | **3** | **1** | **3** | **3** | **2** | **3** | **3** | **2** | **3** |
| **Total Score** | **32** | **38** | **26** | **18** | **30** | **29** | **40** | **30** | **21** | **26** | **38** | **25** | **40** | **38** | **23** | **16** |

*Y=Yes, N=No, U= unclear, NA= Not applicable

**#Table listing all the items of R-AMSTAR**

| **1** | **Was an 'a priori' design provided?** |
| --- | --- |
| **1**(A) | ’a priori’ design |
| **1**(B) | Statement of inclusion criteria |
| **1**(C) | PICO/PIPO research question (population, intervention, comparison, prediction, outcome) |
| **2** | **Was there duplicate study selection and data extraction?** |
| **2**(A) | There should be at least 2 independent data extractors as stated or implied |
| **2**(B) | Statement of recognition or awareness of consensus procedure for disagreements |
| **2**(C) | Disagreements among extractors resolved properly as stated or implied |
| **3** | **Was a comprehensive literature search performed?** |
| **3**(A) | At least 2 electronic sources should be searched |
| **3**(B) | The report must include years and databases used (e.g. CENTRAL, MEDLINE, EMBASE) |
| **3**(C) | Keywords or MESH terms (or both) must be stated AND where feasible the search strategy outline should be provided such that one can trace the filtering process of the included articles |
| **3**(D) | In addition to the electronic databases (PubMed, MEDLINE, EMBASE), all searches should be supplemented by consulting current contents, reviews, textbooks, specialized registers, or experts in the particular field of study, and by reviewing the references in the studies found |
| **3**(E) | Journals were “hand-searched” or “manual searched” (i.e. identifying highly relevant journals and conducting a manual, page by- page search of their entire contents looking for potentially eligible studies) |
| **4** | **Was the status of publication (i.e. grey literature) used as an inclusion criterion?** |
| **4**(A) | The authors should state that they searched for reports regardless of their publication type |
| **4**(B) | The authors should state whether or not they excluded any reports (from the systematic review), based on their publication status, language, etc. |
| **4**(C) | “Non-English papers were translated” or readers sufficiently trained in foreign language |
| **4**(D) | No language restriction or recognition of non-English articles |
| **5** | **Was a list of studies (included and excluded) provided?** |
| **5**(A) | Table/list/figure of included studies, a reference list does not suffice |
| **5**(B) | Table/list/figure of excluded studies, either in the article or in a supplemental source (i.e. online). (Excluded studies refers to those studies seriously considered on the basis of title and/or abstract, but rejected after reading the body of the text) |
| **5**(C) | Author satisfactorily/ sufficiently stated the reason for exclusion of the seriously considered studies |
| **5**(D) | Reader was able to retrace the included and the excluded studies anywhere in the article bibliography, reference or supplemental source |
| **6** | **Were the characteristics of the included studies provided?** |
| **6**(A) | In an aggregated form such as a table, data from the original studies should be provided on the participants, interventions AND outcomes |
| **6**(B) | Provide the ranges of relevant characteristics in the studies analysed (e.g. age, race, sex, relevant socioeconomic data, disease status, duration, severity or other diseases should be reported) |
| **6**(C) | The information provided appears to be complete and accurate (i.e. there was a tolerable range of subjectivity here. Is the reader left wondering? If so, state the needed information and the reasoning) |
| **7** | **Was the scientific quality of the included studies assessed and documented?** |
| **7**(A) | ’A priori’ methods of assessment should be provided (e.g. for effectiveness studies if the author(s) chose to include only randomized, double-blind, placebo controlled studies, or allocation concealment as inclusion criteria); for other types of studies alternative items will be relevant |
| **7**(B) | The scientific quality of the included studies appeared to be meaningful |
| **7**(C) | Discussion/ recognition/ awareness of level of evidence |
| **7**(D) | Quality of evidence should be rated/ranked based on characterized instruments. (Characterized instrument is a created instrument that ranks the level of evidence, e.g. GRADE (Grading of Recommendations Assessment, Development and Evaluation)) |
| **8** | **Was the scientific quality of the included studies used appropriately in formulating conclusions?** |
| **8**(A) | The results of the methodological rigor and scientific quality should be considered in the analysis and the conclusions of the review |
| **8**(B) | The results of the methodological rigor and scientific quality were explicitly stated in formulating recommendations |
| **8**(C) | To have conclusions integrated/ drives towards a clinical consensus statement |
| **8**(D) | This clinical consensus statement drives towards revision or confirmation of clinical practice guidelines |
| **9** | **Were the methods used to combine the findings of studies appropriate?** |
| **9**(A) | Statement of criteria that were used to decide that the studies analysed were similar enough to be pooled? |
| **9**(B) | For the pooled results, a test should be done to ensure the studies were combinable, to assess their homogeneity (i.e. Chi2 test for homogeneity, I2 statistic) |
| **9**(C) | Is there a recognition of heterogeneity or lack of thereof |
| **9**(D) | If heterogeneity exists a “random-effects model” should be used or the rationale (i.e. clinical appropriateness) of combining should be taken into consideration (i.e. is it sensible to combine?), or stated explicitly (or both) |
| **9**(E) | If homogeneity exists, author should state a rationale or a statistical test |
| **10** | **Was the likelihood of publication bias (a.k.a. “file drawer” effect) assessed?** |
| **10**(A) | Recognition of publication bias or filedrawer effect |
| **10**(B) | An assessment of publication bias should include graphical aids (e.g. funnel plot, other available tests) |
| **10**(C) | Statistical tests (e.g. Egger regression test) |
| **11** | **Was the conflict of interest stated?** |
| **11**(A) | Statement of sources of support |
| **11**(B) | No conflict of interest. This is subjective and may require some deduction or searching |
| **11**(C) | An awareness/ statement of support or conflict of interest in the primary inclusion studies |
