# Supplementary material for: Effect of vitamin A, calcium and vitamin D fortification and supplementation on nutritional status of women: an overview of systematic reviews
Source: Syst Rev. 2020 Oct 27;9:248. doi: 10.1186/s13643-020-01501-8 (PMC7592561; doi:10.1186/s13643-020-01501-8)
Supplement: Supplementary file 6 — Additional file 6. References of the primary studies. This file consists of the references for the primary studies or RCTs included in the systematic reviews that were used for data synthesis. [file 13643_2020_1501_MOESM6_ESM.docx]

**Additional file 5: Detail synthesis of food fortification interventions**

**Table 1 Effect of vitamin A supplementation on maternal outcomes**

| 1. Vitamin A supplementation |
| --- |
| 1.1. Serum retinol levels and hepatic reserves in women |
| 1.1.1 Serum retinol levels in women |
| Two Systematic Reviews (SRs) of high (Oliveira et al., 2016), and low quality (Caminha, et al., 2009) reported the vitamin A supplementation and its effect on serum retinol levels. These three SRs considered 11 RCTs that contributed towards this outcome. Four RCTs recruited pregnant women (Ayah 2007; Martins 2010; Roy 1997; Vinutha 2000), one included women of reproductive age group from 13-42 years (Fernandes 2012) and six included postpartum women (RETIBETA project; Rice 1999; Stolzfus 1993; Darboe 2007; Tchum 2006; Zvandsara 2006; Malaba 2005).  Four RCTs (Martins 2010; RETIBETA project; Roy 1997; Vinutha 2000) administered single dose of 200,000 IU vitamin A to the postpartum women in the intervention group. However, time of administering the vitamin A differed between these RCTs; 20-30^th^ day postpartum (Martin 2010), retinyl palmitate within 1-3 weeks postpartum or 7.8 mg of beta carotene daily for nine months (RETIBETA project), retinyl palmitate within 24 hours of delivery in addition to sixty mg of iron daily to the intervention group (Roy 1997) and within 48 hours of delivery orally (Vinutha 2000). One RCT (Stolzfus 1993) provided single high dose of 300,000 IU vitamin A as retinyl palmitate to women within 1-3 weeks after birth.  Three RCTs (Ayah 2007; Zvandsara 2006; Malaba 2005) provided high dose of 400,000 IU to the postpartum women in the intervention group but timing of intervention varied. Retinyl palmitate within 24 hours of delivery (Ayah 2007), in less than 96 hours post-delivery (Zvandsara 2006); and retinyl palmitate to the postpartum period (Malaba 2005).  Three RCTs (Darboe 2007; Fernandes 2012; and Tchum 2006) compared the effects of single dose 200,000 IU vitamin A at delivery to one group and two doses of 200,000 vitamin A, one at delivery and second after a week, to another group.  Oliveira, et al., 2016 included five RCTs (Ayah 2007; Martins 2010; RETIBETA Project; Roy 1997; Stolzfus 1993a) for reporting the outcome. The analysis for different doses of vitamin A and their effect on serum retinol concentration based on different follow up periods and subgroup analysis for different dosages is mentioned below:   1. For follow up period of 3-3.5 months postpartum: 2. Dose 200,000-400,000 IU of vitamin A: Oliveira, et al. 2016 reported that 200,000-400,000 IU dose of vitamin A supplementation when given to postpartum women (n=704) significantly increased the maternal serum retinol levels, in comparison to placebo when assessed at 3-3.5 months (Mean Difference (MD) 0.11 micromol/L, 95% CI 0.03-0.19; I2=39%). Out of five RCTs, three were judged to have low risk of bias (ROB) for allocation concealment and sequence generation and two were judged to be of unclear ROB for these two domains, on the Cochrane ROB tool. 3. Dose 200,000-300,000 IU of vitamin A: Oliveira, et al., 2016 reported the effect of administration of 200,000-300,000 IU of vitamin A as supplementation versus placebo, for postpartum women and outcome was assessed at 3-3.5 months postpartum. A significant increase of the maternal serum retinol levels after intervention was reported (MD 0.17 micromols 95% CI 0.07, 0.26; I2=0.0%; four RCTs) (Martins 2010; RETIBETA Project; Roy 1997; Stozfus 1993a). 4. Dose of 400,000 IU of vitamin A: It was reported that administration of one single dose of 400,000 IU within 24 hours of birth to women had no significant increase in the maternal serum retinol levels (0.04MD micromoles/L, 95% CI -0.01, 0.09), in comparison to placebo; when assessed at 3-3.5 months postpartum. This outcome was assessed by one RCT (Ayah 2007) with 402 participants that scored low ROB for five domains in the Cochrane ROB tool (Oliveira, et al., 2016). 5. Daily administration of 7.8 mg beta carotene postpartum: One RCT (RETIBETA Project) administered daily 7.8 mg beta carotene to postpartum women (n=54). It was found that intervention did not significantly increase the serum retinol levels (MD 0.10 µmol/L, 95% CI -0.14 to 0.34), in comparison to a placebo; when assessed at 3-3.5 months postpartum (Oliveira, et al., 2016). 6. For follow-up period of 6-6.5 months postpartum 7. Dose 200,000-400,000 IU vitamin A: It was reported that maternal serum retinol concentration was not enhanced for 200,000-400,000 IU of vitamin A supplementation (MD 0.06, 95% CI -0.06 to 0.18; I2=62%). The result is generated by including four RCTs (Ayah 2007; RETIBETA Project; Roy1997; Stolzfus 1993a), with 533 participants (Oliveira, et al., 2016). 8. Single Dose of 400,000 IU vitamin A: No increase in maternal serum retinol concentration (MD -0.02, 95% CI -0.08 to 0.04). The result is identified from one RCT (Ayah 2007), with 291 participants (Oliveira, et al., 2016). 9. Daily administration of 7.8 md beta carotene postpartum: No increase in serum retinol levels (MD 0.05, 95% CI -0.28 to 0.38) in comparison to placebo when assessed at 6-6.5 months postpartum. The result is from one RCT (RETIBETA Project) with 50 participants (Oliveira, et al., 2016). 10. Dose 200,000-300,000 IU vitamin A: A significant increase in maternal serum retinol levels were reported with 200,000-300,000 IU vitamin A supplementation (MD 0.13, 95% CI 0.03 to 0.23; I2=0%), in comparison to placebo; when assessed at 6-6.5 months postpartum. The result was identified from three RCTs (RETIBETA project; Roy 1997; Stolzfus 1993a), with 242 participants (Oliveira, et al., 2016). 11. For follow-up period nine months postpartum 12. Dose 200,000-300,000 IU vitamin A: The serum retinol levels did not show any significant difference by administering 200,000-300,000IU of vitamin A as compared to placebo (MD 0.00, 95% CI -0.16 to 0.17). Two RCTs (RETIBETA Project; Roy 1997) contributed for this outcome by including 98 participants (Oliveira, et al., 2016). 13. 7.8 mg daily dose of beta-carotene: No significant difference seen on outcome when assessed at nine months postpartum women (n-51) (MD 0.19, 95% CI -0.08 to 0.46 (RETIBETA Project) (Oliveira, et al., 2016).   It was reported (Darboe 2007; Fernandes 2012) that a dose of 400,000 IU vitamin A did not change the mean maternal serum retinol levels significantly when assessed at two (MD 0.04 95% CI -0.06, 0.14; 429 participants), four (MD -0.12 95% CI -0.29, 0.05; 213 participants) or six months (MD -0.08 95% CI -0.24, 0.08; 200 participants); in comparison to the standard dose of 200,000 IU. The two RCTs were scored as low ROB for four and six domains (both including allocation concealment and sequence generation), out of seven domains in the Cochrane ROB tool (Oliveira, et al., 2016).  Another SR (Caminha, et al., 2009), reported vitamin A supplementation and its effect on maternal serum retinol levels. The analysis for different follow-up periods and different dosages is categorized below:   1. For follow-up period 2.5 months postpartum and dose 200,000 IU vitamin A: A high dose of 400,000 IU vitamin A did not significant affect on the maternal serum retinol levels, when compared to a standard dose of 200,000 IU at 2-5 months postpartum. The result was identified from two RCTs (Darboe et. Al 2007; Tchum et.al 2006), with 387 participants. 2. For follow-up period of three months postpartum and dose 200,000 IU vitamin A: Three RCTs assessed the effects of 200,000 IU vitamin A on maternal serum retinol levels. It was reported that when 200,000 IU of vitamin A is administered up to 48 hours (Vinutha et.al 2000) and 15 days (Rice et.al 1999) after delivery, it has no effect on the maternal serum retinol levels; when assessed at three months after the delivery. The number of participants in the two RCTs were 256 women. 3. For follow-up period of six months postpartum 4. Dose 200,000 IU vitamin A: A dose of 200,000 IU of vitamin A administered up to 24 hours after delivery elevated the serum retinol by 0.26 micromol/l; when assessed at six month postpartum, identified from one RCT (Roy et.al 1997) with 50 participants. 5. Dose 300,000 IU vitamin A: Another RCT (Stolzfus et.al 1993), with 153 participants found that administration of 300,000 IU of vitamin A elevated the serum retinol levels by 0.15 micromol/l; when assessed at six months postpartum. This RCT was scored as low risk on ROB for four domains (including allocation concealment and sequence generation), out of seven domains. 6. Dose 400,000 IU vitamin A: Three RCTs (Ayah et. al 2007; Zvandsara et.al 2006; Malaba et.al 2005) assessed the effect of 400,000 IU vitamin A on the maternal serum retinol levels, in comparison to a placebo. One RCT (Ayah et. al 2007), with 564 participants found that 400,000 IU vitamin A given 24 hours after delivery had no effect on serum retinol levels; when assessed at six months postpartum. Two large RCTs consisting of 14110 (Zvandsara et.al 2006) and 8808 (Malaba et.al 2005) participants found that administration of 400,000 IU vitamin A, up to 96 hours after delivery, elevates the serum retinol levels by 0.44 micromol/l (Zvandsara et.al 2006) and 0.16 micromol/l (Malaba et.al 2005); in comparison to a placebo; when assessed after 45 days. 7. For follow-up period nine months postpartum and dose 200,000 IU vitamin A: Two RCTs found that administration of 200,000 IU of vitamin A up to 48 hours (Vinutha et.al 2000) and 15 days (Rice et.al 1999) after delivery has no effect on the maternal serum retinol levels at nine months postpartum. |
| 1.1.2 Hepatic reserves in women |
| Oliveira, et al 2016, including two RCTs (RETIBETA project; Stolzfus 1993) contributed to this outcome. Participants were postpartum women and dose of 200,000 -300,000 IU vitamin A and 7.8 mg beta carotene daily was given to the participants.  The analysis for different follow up periods and subgroup analysis for different dosages is mentioned below:   1. For follow-up period three months postpartum: The hepatic reserves for vitamin A were not significantly increased for proportion of women after supplementation of a single dose of 200,000-300,000 vitamin A (RR 0.58, 95% CI 0.16 to 2.08; I2=73%), in comparison to placebo; when assessed at three months. The outcome was assessed by two RCTs (RETIBETA project; Stolzfus 1993a) with 191 participants. The statistical heterogeneity for this outcome was high I^2^=73%. Both the RCTs scored four domains as low risk (including allocation concealment and sequence generation), out of seven domains of a Cochrane ROB tool (Oliveira, et al., 2016). 2. For follow-up period six months postpartum: The results were not significant with same dosage even when the hepatic reserves were assessed at six postpartum (RETIBETA project; Stolzfus 1993a). 3. For follow-up period nine months postpartum: The results were not significant with 200,000-300,000 IU vitamin A even when the hepatic reserves were assessed at nine months postpartum (RETIBETA project; Stolzfus 1993a).   Daily supplementation of 7.8 mg of beta-carotene did not report a significant increase in the hepatic reserves ((RR 0.59, 95% CI 0.27 to 1.30; assessed using one RCT (RETIBETA project) with 51 participants in comparison to placebo; when assessed at nine months postpartum. |
| 1.2. Vitamin A contents in breast milk, proportion of women with low vitamin A contents in breast milk and sIgA levels in colostrum |
| - - 1. Vitamin A contents in breast milk |
| Three SRs of high quality (Oliveira, et al., 2016); moderate quality (Neves, et al., 2015) and low quality (Caminha, et al., 2009) reported the effect of vitamin A supplementation on breast milk retinol concentration or vitamin A contents in breast milk. These three SRs included sixteen RCTs (Dimenstein et. al 2007; Basu 2003; Bahl 2002; Vinutha 2000; Rice 1999; Bhaskaram 1998; Roy 1997; Stozfus 1993; Idindili 2007; Darboe 2007; Ayah 2007; Bezerra 2009; Bezerra 2010; Ribeiro 2009; Martin 2010; WHO/CHD IVASSG; Fernandes 2012).  Participants were pregnant women (Ayah 2007; Martin 2010; Roy 1997; Basu 2003) and postpartum women (Stolzfus 1993; RETIBETA project; Darboe 2007; Fernandes 2012; Vinutha 2000; Dimenstein 2007; Bhaskaram 1998; Idindili 2007; Bezerra 2009; Bezerra 2010; Ribeiro 2009; WHO/CHD IVASSG).  Intervention:  Single dose of 200,000 IU vitamin A as retinyl palmitate supplements in the postpartum (Martin 2010; RETIBETA Project; Roy 1997; Vinutha 2000; Dimenstein 2007; Bahl et al 2002; Basu 2003; Bhaskaram 1998; Bezerra 2009; Ribeiro 2009; Rice et. al 1999; WHO/CHD IVASSG); vitamin A supplements were given up to 12 hours after the delivery (Dimenstein 2007); 24 hours after delivery (Basu 2003; Bhaskaram 1998) and within 18-42 days after delivery (WHO/CHD IVASSG project). One RCT (Stolzfus 1993) provided single high dose of 300,000 IU vitamin A as retinyl palmitate to women within 1-3 weeks after birth. Five RCTs (Ayah 2007; Darboe 2007; Fernandes 2012; Idindili 2007; Bezerra 2010) provided 400,000 IU vitamin A supplements.  Time of breast milk collection: morning (Dimenstein 2007; Basu 2003; Bhaskaram 1998; Roy 1997; Stolzfus 1993; Bezerra 2009; Bezzerra 2010); early morning to noon time (WHO/CHD IVASSG and Vinutha 2000), morning till evening (RETIBETA project) and not stated (Idindili 2007; Darboe 2007; Ayah 2007).  Technique of breast milk collection: either manual expression or manual pump (Dimenstein 2007; Basu 2003; Vinutha 2000; WHO/CHD IVASSG; RETIBETA project; Stolzfus 1993; Darboe 2007; Ayah 2007; Bezerra 2009; Bizerra 2010) and not stated (Bhaskaram 1998; Roy 1997; Idindili 2007; Ribeiro 2009; Fernandes 2012; Martins 2010).  High performance liquid chromatography was used by most of the RCTs as the method for analysis of retinol contents in the breast milk.  Meta-analysis was performed by only one SR (Oliveira, et al., 2016), the other SRs (Neves, et al., 2015; Caminha, et al., 2009) presented the results in the narrative format. Only one SR (Oliveira, et al., 2016) used GRADE tool for assessing the quality of evidence of this outcome for the effect of 200,000-400,000 IU vitamin A on retinol levels of breast milk, and it was reported to be of low quality. Quality of the included RCTs was heterogeneous.  Oliveira, et al., 2016 (high quality SR), reported concentration of retinol in breast milk and proportion of women with low breast milk retinol contents. The analysis for the effect of different doses of vitamin A on breast milk retinol concentration based on different follow up periods and subgroup analysis for different dosages is mentioned below.   1. For follow-up period 3-3.5 months postpartum 2. Dose 200,000-400,000 IU vitamin A: Supplementation with 200,000-400,000 IU of vitamin A to women increased the retinol levels in breast milk (MD 0.20 µmol/L, 95% CI 0.08 to 0.3; I2=58%), in comparison to placebo or no treatment, when assessed at three or three and a half month postpartum (Ayah 2007; Martins 2010; RETIBETA project; Roy 1997; Stolzfus 1993a; Vinutha 2000) with total 837 participants and the quality of evidence for this outcome was reported to be low, as per the GRADE assessment scale. 3. Dose 200,000-300,000 IU vitamin A: It was reported that, with dose of 200,000-300,000 IU of vitamin A, the effect on retinol levels in breast milk was significantly increased (MD 0.25 µmol/L, 95% CI 0.12 to 0.37; I2=21%), in comparison to placebo or no treatment; when assessed at three to three and a half months postpartum. Five RCTs (Martins 2010; RETIBETA project; Roy 1997; Stolzfus 1993a; Vinutha 2000) contributed to the result of this outcome, with total 415 participants. The evidence quality was assessed to be of low on the GRADE assessment scale for this outcome. 4. Single dose of 400,000 IU vitamin A: A single dose of 400,000 IU of vitamin A reported an increase in the mean levels of retinol concentration in breast milk (MD 0.08 95% CI 0.03, 0.13), in comparison to placebo or no treatment; when assessed at three-three and half months postpartum. However, this outcome was assessed by a single RCT (Ayah 2007) that scored five domains as low ROB (including allocation concealment and sequence generation), out of seven domains in the Cochrane ROB assessment tool. 5. Daily supplementation with 7.8 mg beta carotene: Supplementation with daily 7.8 mg of beta carotene during for nine months postpartum, did not have a significant effect on the breast milk retinol levels (MD 0.02 95% CI -0.17, 0.2), in comparison to placebo or no treatment; when assessed at 3-3.5 months postpartum (RETIBETA project).   The sub group heterogeneity for assessing breast milk retinol concentration at 3-3.5 months was high (I^2^=67%), after sensitivity analysis this was reported to be due to Ayah 2007 and Stolzfus 1993a that used higher doses of vitamin A.   1. For follow-up period of six months postpartum   Contrary to the above results, the effect of vitamin A supplementation on breast milk concentration did not report any significant changes when assessed at six months postpartum.   - 1. Dose 200,000-400,000 IU vitamin A: Four RCTs (Ayah 2007; RETIBETA project; Roy 1997; Stolzfus 1993a), with 645 participants found that supplementation with 200,000-400,000 IU of vitamin A to postpartum women did not have any significant effect on the breast milk retinol concentration (MD 0.20 95% CI -0.01, 0.41; I^2^=80%), in comparison to a placebo or no treatment.   2. Dose 200,000-300,000 IU vitamin A: Three RCTs (RETIBETA project; Roy 1997; Stolzfus 1993a) with 291 participants found that when 200,000-300,000 IU of vitamin A is supplemented to postpartum women, it results in no significant changes in the breast milk retinol concentrations (MD 0.28 95% CI -0.04, 0.60; I^2^=78%), in comparison to a placebo or no treatment (Oliveira, et al., 2016).   3. Single dose of 400,000 IU vitamin A: One RCT (Ayah 2007) with 354 participants found that administration of a single high dose of 400,000 IU to postpartum women did not have any effect on the breast milk retinol concentration (MD 0.06 95% CI 0.01, 0.11), in comparison to a placebo or no treatment (Oliveira, et al., 2016).   4. Daily dose of 7.8 mg beta carotene: RETIBETA project concluded that supplementation with 7.8 mg daily dose of beta carotene for nine months did not have any significant effect on the breast milk retinol concentration (MD 0.12 95% CI -0.13, 0.37) in comparison to a placebo or no treatment; when assessed at six months postpartum (Oliveira, et al., 2016).  1. For follow-up period of nine months postpartum    - 1. Dose 200,000-300,000 IU vitamin A: Three RCTs (RETIBETA project; Roy 1997; Stolzfus 1993a), with 275 participants, assessed the effect of vitamin A supplementation on breast milk retinol concentration at nine months postpartum. It was reported that 200,000-300,000 IU of vitamin A supplementation when administered to women did not significantly effect the breast milk retinol concentration of mothers (MD 0.15 95% CI -0.16, 0.45; I^2^=62%), in comparison to a placebo or no treatment; when assessed at nine months postpartum (Oliveira, et al., 2016).      2. Daily dose of 7.8 mg beta carotene: One RCT (RETIBETA Project) with 103 participants found that supplementation with 7.8 mg daily dose of beta-carotene for nine months, significantly affected the breast milk retinol concentration levels (MD 0.21 95% CI 0.01, 0.41), in comparison to a placebo or no treatment; when assessed at nine months postpartum. Quality assessment for this RCT demonstrated four low ROB domains (including allocation concealment and sequence generation), out of seven domains (Oliveira, et al., 2016).   *Comparison of dose 400,000 IU vitamin A with standard dose of 200,000 IU and its effect on breast milk retinol concentration:*  Oliveira, et al., 2016 reported the effect of 400,000 IU vitamin A on breast milk retinol concentration when compared to a standard dose of 200,000 IU at two and four months. No significant effect of 400,000 IU vitamin A was reported on the level of maternal breast milk retinol concentrations (MD -0.01 95% CI -0.14, 0.11;I2=0%, n=377), in comparison to 200,000 IU vitamin A assessed at two months postpartum or at four months postpartum (MD -0.03 95% CI -0.13, 0.08; I2=0%, n=348). Both the RCTs (Darboe 2007; Fernandes 2012) were scored as low ROB for four and six domains (both including allocation concealment and sequence generation), out of seven domains in the Cochrane ROB tool.  Oliveira, et al., 2016 reported the effect of 400,000 IU vitamin A on maternal breast milk retinol concentration, in comparison to a standard dose of 200,000 IU. The effect of 400,000 IU vitamin A on maternal breast milk retinol concentration at three months postpartum (MD -0.06 95% CI -0.28, 0.16; n=110) or at six months postpartum (MD 0.09 95% CI -0.13, 0.31; n=192); was not significant. The RCT (Darboe 2007) included for analysis was scored as low ROB for six domains (including allocation concealment and sequence generation), out of seven on the Cochrane ROB assessment tool. |
| - - 1. Proportion of women with low vitamin A contents |
| Oliveira, et al., 2016 assessed the effect of vitamin A on proportion of women having low breast milk retinol concentrations. The analysis based on different follow up periods and dosages for the same, is given below.  For follow-up period of three months postpartum   1. Dose 200,000-300,000 IU vitamin A: Three RCTs (RETIBETA Project, Stolzfus 1993a; Vinutha 2000), with 304 participants found that proportion of women with low breast milk retinol concentration was significantly reduced after supplementation with a single dose of 200,000-300,000 IU of vitamin A (RR 0.56, 95% C I 0.37 to 0.84; I^2^=55%), in comparison to a placebo or no treatment; when assessed at three months postpartum. Two RCTs (RETIBETA Project; Stolzfus 1993)for this outcome found to be having four domains as low ROB (including allocation concealment and sequence generation), whereas one had only one domain as low risk with allocation concealment and sequence generation as unclear ROB (Oliveira, et al., 2016). 2. Single dose of 200,000 IU vitamin A: One RCT (RETIBETA Project) with 105 participants found the effect of administering a single dose of 200,000 IU vitamin A on the proportion of women with low breast milk retinol concentration (< 0.28 µmol/g of fat), at three months postpartum (Oliveira, et al., 2016). 3. Daily dose of 7.8 mg beta carotene: One RCT (RETIBETA Project), assessed the effect of 7.8 mg daily dose of beta carotene. The supplementation did not show any significant effect when assessed at three months (RR 1.01 95% CI 0.79, 1.29; 109 participants) (Oliveira, et al., 2016).   For follow-up period of six months postpartum  a) 200,000 IU Vitamin A: Two RCTs (RETIBETA project and WHO/CHD IVASSG), found that there was no significant effect on the proportion of women with low breast milk retinol concentration (< 0.28 µmol/g of fat), when assessed at six months (RR 0.85 95% CI 0.72, 1.01; 779 participants) (Oliveira, et al., 2016).  b) Daily dose of 7.8 mg beta carotene: One RCT (RETIBETA Project), assessed the effect of 7.8 mg daily dose of beta carotene on the proportion of women with low breast milk retinol concentration (< 0.28 µmol/g of fat), at six months (RR 0.88 95% CI 0.69, 1.12; 104 participants) (Oliveira, et al., 2016).  For follow-up period of nine months postpartum   1. Dose 200,000 IU vitamin A: Two RCTs (RETIBETA project and WHO/CHD IVASSG), found that there was no significant effect on the proportion of women with low breast milk retinol concentration (< 0.28 µmol/g of fat), when assessed at nine months postpartum (RR 0.87 95% CI 0.73, 1.04; 667 participants). Both the RCTs were judged to have seven and four low ROB domains out of seven on a Cochrane ROB assessment tool (Oliveira, et al., 2016). 2. Daily 7.8 mg beta carotene: One RCT (RETIBETA Project), assessed the effect of 7.8 mg daily dose of beta carotene for nine months postpartum on the proportion of women with low breast milk retinol concentration (< 0.28 µmol/g of fat) and demonstrated an improvement at nine months postpartum (RR 0.76, 95% CI 0.60 to 0.97; 102 participants). The RCT reported to be having four domains as low ROB out of seven in the quality assessment tool (Oliveira, et al., 2016).   *Comparison of dose 400,000 IU vitamin A with standard dose of 200,000 IU and its effect on proportion of women with low breast milk retinol concentration:*  Oliveira, et al., 2016, reported the effect of 400,000 IU vitamin A on the proportion of women with low breast milk retinol concentration, in comparison to 200,000 IU of vitamin A. It was reported that there was no significant effect of 400,000 IU vitamin A supplementation on the proportion of women with low breast milk concentration when assessed at three months postpartum (RR 1.25 95% CI 0.72, 2.17; n= 110) and six months postpartum (RR 0.81 95% CI 0.53, 1.25; n=112). The RCT (Darboe 2007) included for analysis was scored as low ROB for six domains (including allocation concealment and sequence generation), out of seven on the Cochrane quality assessment tool.  A moderate methodological quality SR (Neves, et al., 2015), assessed the effects of vitamin A supplementation on the breast milk contents. Two RCTs (Bezerra et.al 2009; Ribeiro et.al 2009) consisting of 85 and 91 women participants each, found an increase in the retinol contents of breast milk after administration of 200,000 IU of vitamin A immediately and sixteen hours after delivery; when followed up after 24 hours of supplementation. Both the RCTs scored less than 3 in the Jadad scale for assessment of quality. Another RCT (Martins et.al 2010) (score <3 on Jadad scale) with 61 puerperal women found that, on administration of 200,000 IU of vitamin A within 20-30 days after delivery, the retinol contents in the breast milk is increased, it reduces the vitamin A deficiency (VAD) prevalence in breast milk to 16% when compared to a 55.6% in the control group. One RCT (Bezerra et.al 2010) (Score <3 on Jadad scale) with 199 women participants found that supplementation with a 200,000 IU immediately after postpartum and an additional second dose of 200,000IU after 24 hours increased the breast milk retinol contents for puerperal women, in comparison to a single dose of 200,000 IU or control; when assessed four weeks after delivery (Neves, et al., 2015).  A low quality SR (Caminha, et al., 2009), assessed the effects of mega doses of vitamin A (i.e. 200,000 IU; 300,000 IU; and 400,000 IU) on the serum and breast milk retinol concentration for postpartum women. The SR included eleven RCTs (Dimenstein et. al 2007; Basu et.al 2003; Bahl et. al 2002; Vinutha et.al 2000; Rice et. Al 1999; Bhaskaram & Balakrishna 1998; Roy et. al 1997; Stozfus et al 1993; Idindili et al 2007; Darboe et al 2007; Ayah et.al 2007), that assessed the effects of vitamin A supplementation on breast milk retinol. Pooled evidence for effectiveness of vitamin A supplementation based on different doses (different RCTs) is given below.   - - 1. For a dose of 200,000 IU: Caminha, et al., 2009 included seven RCTs (Dimenstein et. al 2007; Basu et.al 2003; Bahl et. al 2002; Vinutha et.al 2000; Rice et. Al 1999; Bhaskaram & Balakrishna 1998; Roy et. al 1997) and reported that administration of 200,000 IU of vitamin A given at 12 hours-42 days after delivery (range for different RCTs) elevated the breast milk retinol concentration by 0.17-1.88 micromol/L (range for different RCTs), when assessed at six hours to six months period postpartum (range for different RCTs). The number of participants in these seven RCTs varied from 50-2990 women. The method of breast milk expression was by manual expression or manual pump (Dimenstein et. al 2007; Basu et.al 2003; Bahl et. al 2002; Vinutha et.al 2000; Rice et. al 1999), and no information stated (Bhaskaram & Balakrishna 1998; Roy et. al 1997). Breast milk was expressed from both the breasts (Basu et.al 2003; Bahl et. al 2002; Vinutha et.al 2000), either right or left breast was selected (Dimenstein et. al 2007; Rice et. al 1999), only left breast was preferred (Bhaskaram & Balakrishna 1998) and not reported (Roy et. al 1997). The time for breast milk sample collection was morning in all the RCTs and one RCT (Dimenstein et. al 2007) mentioned that high performance liquid chromatography was used for identification of retinol concentration.     2. For a dose of 300,000 IU vitamin A: It was reported by Caminha, et al., 2009 by including one RCT (Stolzfus et al 1993) that, 300,000 IU of vitamin A given from 7-21 days after delivery, elevated the breast milk retinol levels by 0.48 micromol/l, when assessed after eight months. The time of breast milk collection was morning and by manual pump method. Either right or left breast was used for collection of breast milk after more than one hour post breastfeeding. The number of participants in the RCT were 153 and it scored low ROB for four (including allocation concealment and sequence generation) out of seven domains in the Cochrane tool.     3. For a dose of 400,000 IU v/s 200,000 IU vitamin A: Caminha, et al., 2009 assessed the effect of 400,000 IU vitamin A on the breast milk retinol levels, in comparison to a dose of 200,000 IU vitamin A by including n=1000 (Idindili et al 2007; Darboe et al 2007). It was reported that there was no additive effect of a dose of 400,000 IU when administered within seven days to one month after delivery, on the breast milk retinol levels, when compared to a single standard dose of 200,000 IU vitamin A; assessed at six and nine months postpartum. Time of breast milk collection was not stated by any of the RCTs, and one RCT (Darboe et al 2007) stated manual expression and both the breasts as the preferred choice for the sample collection. Both the RCTs were scored as low risk for allocation concealment and sequence generation on the Cochrane tool.     4. For 400,000 IU vs placebo: Caminha, et al., 2009 by including one RCT (Ayah et. al 2007) with 564 participants, assessed the effect of 400,000 IU vitamin A on the breast milk retinol concentration, administered 24hrs after delivery. There was elevation in the breast milk retinol levels by 0.06 micromol/l, in comparison to placebo; when assessed at six months postpartum. The time for the breast milk collection was not sated, but it was carried out using the manual expression technique, one hour after breastfeeding. |
| - - 1. Secretary immunoglobulin A (sIgA) levels in colostrum |
| Neves, et al., 2015 included one RCT (Lima et. al 2012) (score<3 on Jadad scale) with 96 mothers to assess the effect of vitamin A supplementation on secretory immunoglobulin A (sIgA) content in colostrum. It was reported that administration of 200,000 IU of retinoyl palmitate (vitamin A) supplement immediately after delivery significantly increases the sIgA content of the colostrum, in comparison to the control group; when assessed after 24 hours of the supplementation. |
| 1.3 Clinical and Subclinical Vitamin A deficiency & Night Blindness |
| Two high quality SR (Oliveira, et al., 2016; Mc Cauley, et al., 2015) reported the effects of vitamin A supplementation on clinical and subclinical vitamin A deficiency and night blindness.  1.3.1. Abnormal conjunctival impression cytology (CIC): Oliveira et al., 2016, included one RCT (Stolzfus 1993), reported the effect of administering single high dose of 300,000 IU vitamin A at 2-3 weeks after delivery, on the proportion of women having abnormal CIC. There was no difference reported at three months (RR 1.00 95% CI 0.55, 1.80; n=148) and six months (RR 0.56 95% CI 0.27, 1.17; n=142), when compared to a placebo or no treatment. This RCT reported four domains for low ROB (including allocation concealment and sequence generation) out of seven.  1.3.2. Risk of night blindness: McCauley, et al., 2015 reported that supplementation with vitamin A reduced the risk of night blindness among pregnant women (RR 0.79 95% CI 0.64, 0.98; I2=85%) when compared to placebo or no treatment, this was assessed by two large cluster randomized RCTs (West 1999; West 2011) with 10,068 participants. The dose of vitamin A used was 7000 mcg of retinol equivalents. |
| 1.4. Maternal anaemia and haemoglobin |
| One medium quality (Thorne Lyman & Fawzi, 2012) SR reported this outcome. The Quality of the included RCTs was heterogeneous.  Thorne-Lyman & Fawzi, 2012 included eight RCTs (Ma 2008; Suharno 1993; Van der Broek 2006; Muslimatum 2001; Radhika 2003; Semba 2001; Cox 2005; and Dijkhuizen 2004) that contributed towards this outcome. Participants were pregnant women of not more than 24 weeks gestation, except one RCT (Semba 2001), which included pregnant women of 18-28 weeks gestation. Status of anaemia: anaemic pregnant women (Cox 2005; Muslimatum 2001; Suharno 1993; Van der Broek 2006; Ma 2008), non-anaemic women (Dijkhizen 2004) and information about anaemia status was not available (Radhika 2003; Semba 2001).  Intervention: A dose of 5750 IU vitamin A daily (Dijkhuizen 2004) and 20,000 IU weekly (Muslimatum 2001) to the pregnant women. A dose of 3000 IU (Radhika 2003), 10,000 IU (Semba 2001) and 8000 IU (Suharno 1993) of vitamin A was provided for 8 weeks daily until delivery to the pregnant women. One RCT provided a dose of 5000 IU and 10,000 IU to two groups of pregnant women daily for eight weeks until delivery (Van der Broek 2006). One RCT (Ma 2008) provided 2 mg and 2000 microgram retinol daily to two groups of pregnant women and one RCT (Cox 2005) provided 10,000 IU for six weeks to the pregnant women. For all the RCTs except one (Dijkhuizen 2004), levels of vitamin A were measured before initiation of the supplementation.  Thorne-Lyman & Fawzi, 2012 performed following meta-analysis:   1. Effect of vitamin A on risk of anaemia for anaemic women (Hb<11g/dl) at baseline: Thorne-Lyman & Fawzi, 2012 included three RCTs (Ma 2008; Suharno 1993; Van Den Broek 2006), which individually reported a positive effect of vitamin A supplementation and generated a pooled effect estimate of (RR 0.73 95%CI 0.54, 0.99; I^2^ 72%). The heterogeneity reported in the effect estimate was corrected upon removal of particular intervention and control arms of an RCT (Suharno 1993) during sensitivity analysis (RR=0.86 95% CI 0.79, 0.93; I^2^=0%). 2. Effect of vitamin A on risk of anaemia on both anaemic and non-anaemic pregnant women: Thorne-Lyman & Fawzi, 2012 by including three RCTs (Muslimatum 2001; Radhika 2003; Semba 2001) reported that vitamin A has a significant effect on risk of anaemia (RR 0.83 95% CI 0.74, 0.94; I2=0%). 3. Effect of vitamin A on severe anaemia: Thorne-Lyman & Fawzi, 2012 by including two RCTs (Ma 2008;Van Den Broek 2006) found a pooled estimate (RR 0.93 95% CI 0.59, 1.45; I2=0%) and reported there was no significant effect of the intervention in pregnant women. 4. Effect of vitamin A on maternal haemoglobin levels: Thorne-Lyman & Fawzi, 2012 included three RCTs (Cox 2005; Dijkhuizen 2004; Muslimatun 2001) and reported no difference between vitamin A supplemented group and control on maternal haemoglobin levels when assessed at 4-6 months postpartum (MD 0.01 95% CI -0.24, 0.25; I2=0%). |

| **Table 2: Effects of Vitamin D and calcium supplementation and fortification on important maternal outcomes** |
| --- |
| **2.1 Vitamin D supplementation** |
| 2.1.1 25 hydroxy vitamin D levels: |
| Five SRs assessed 25-hydroxyvitamin D (25(OH)D) levels in women of which two had good methodological quality (Regil et al., 2016; Roth et al., 2017) and rest had moderate methodological quality (Chakhtoura et al., 2017; Christesen et al., 2012; Perez-Lopez et al., 2015). All SRs reported that Vitamin D supplementation had beneficial effect on 25(OH)D when compared to placebo/no intervention/low dose vitamin D group, in pregnant women at the time of delivery.  About 41 publications of RCTs were identified from Iran (Abotorabi 2017; Asemi 2013a and b; Asemi 2012; Etemadifar 2015; Hashemipour 2014; Hashemipour 2013; Karamali 2015; Mojibian 2015; Sabet 2012; Samimi 2015; Soheilykhan 2013; Valizadeh 2016; Vaziri 2016; Yazdchi 2016), UAE (Dawodu 2013), Bangladesh (Roth 2013; Islam 2010), India (Sablok 2015; Sahoo 2016; Sahu 2009), Pakistan (Khan 2016), Brazil (Diogenes 2013), Australia (Rodda 2015; Yap 2014), New Zealand (Grant 2014; Grant 2013), UK (Brooke 1980; Cooper 2016; Yu 2009; Yu 2008), USA (Hollis 2011; Litonjua 2016; Thiele 2016; Zerofsky 2014), Canada (March 2015), Denmark (Chawes 2016), Turkey (Mutlu 2014) and France (Delvin 1986; Mallet 1986). Risk of bias of included RCTs was heterogeneous.  A moderate methodological quality SR (Perez-Lopez et al., 2015) conducted a meta-analysis using eight RCTs of which two (Holis 2011; Yu 2009) were three arm RCTs. Included number of pregnant women ranged from 20 to 169 (total n=732) in intervention group, and 20 to 166 (total n=736) in control group. Year of publication of included RCTs ranged from 1980 to 2014. Three RCTs were conducted in Iran (Asemi 2013; Hashemipour 2014; Sabet 2012), two in UK (Brooke 1980; Yu 2009), one each in USA (Holis 2011), France (Delvin 1986) and Bangladesh (Roth 2013). Mean ages ranged from 22.4-27.6 in control group and 22.4-27.4 in treatment group. Age of participants from two RCTs (Delvin 1986; Yu 20009) was not provided. Gestational age at sampling ranged from 12-16 to 28-32 weeks. ROB measured using Cochrane ROB tool was found to be heterogeneous.  Four RCTs had a control group as placebo (Asemi 2013; Brooke 1980; Roth 2013; Sabet 2012), two had no intervention (Delvin 1986; Yu 2009), one RCT had elemental calcium and multivitamin (Hashemipour 2014) and one RCT had placebo and multivitamin (Hollis 2011). Intervention was heterogeneous, the details are as follows:  Type and dose:   - D_2_ with a dose of 1000 IU/day (Brooke 1980) and 800 IU/day in one arm and 200,000 IU bolus in another arm (Yu 2009) - 25(OH)D 400 IU (Asemi 2013) - Rest all RCTs used D_3_ 1000 IU/day - 50,000 IU/week along with elemental calcium and multivitamin (Hashemipour 2014) - Multivitamin along with 1600 and 3600 IU/day of vitamin D in two separate arms (Hollis 2011) - 35,000 IU/week of vitamin D (Roth 2013) - 100,000 IU/mont (Sabet 2012).   Intervention duration ranged from minimum of eight weeks (Hashemipor 2014) to 24-28 weeks (Hollis 2011) in all RCTs. RCTs used different 25(OH)D quantification methods of which only one (Roth 2013) used high performance liquid chromatography which is considered the gold standard method (Perez-Lopez et al., 2015).  Mean difference at delivery using fixed effect measure (FEM) was found to be 66.46 (95% CI 66.22, 66.71; I^2^=100%) favouring intervention group. However, SR authors were unable to conclude about the optimum vitamin D dose during pregnancy owing to heterogeneous frequency, duration and dose of Vitamin D supplementation. It was reported that, included RCTs were of varied methodological quality, did not control the confounding variables (such as body weight, diet, skin characteristics, seasonality and ethnicity) and outcome was not measured uniformly using gold standard essays (Perez-Lopez et al., 2015).  A moderate methodological quality SR (Christesen et al., 2012) included six RCTs conducted in UK (Brooke et al., 1980; Yu et al., 2009), France (Mallet et al., 1986; Delvin et al., 1986) India (Sahu et al., 2009) and USA (Hollis et al., 2011). There were a total of 857 pregnant women but the number of women in each study ranged from 40 to 350. Intervention details are as follows; D_2_ 1000 IU/day during third trimester (Brooke 1980); 1000 IU vitamin D/day in one arm during third trimester and 200,000 IU single dose at seventh month (Mallet 1986); D_3_ 1000 IU/d during third trimester (Delvin et al., 1986); D_3_ 120,000 IU two times at 5^th^ and 7^th^ month and in second arm 60000 IU at 5^th^ month (Sahu 2009); D_3_ 200,000 IU single dose at 27 weeks of gestation in one arm and D_2_ 800 IU/d from week 27 in another arm (Yu 2009); D_3_ 4000 IU/day in one arm and 2000 IU/day in another arm (Hollis 2011). All RCTs compared the intervention to that of no intervention except one (Hollis 2011) that provided 400 IU D3 /d from week 12. SR authors reported that only two trials had high methodological quality (Yu et al., 2009 and Hollis et al., 2011) but not clear how it was assessed. Authors narratively concluded that there was an increase in 25OhD at delivery compared to lower doses or no supplementation.  A good quality SR (Regil et al., 2016) had included 10 RCTs (Asemi 2013; Asemi 2012; Brooke 1980; Delvin 1986; Diogenes 2013; Grant 2013; Mallet 1986; Roth 2013; Sablok 2015; Yu 2008;) that provided information on 25(OH)D levels. Number of participants ranged from 40-260 and were recruited at various weeks of gestation starting from third month to last trimester. One RCT (Diogenes 2013) included adolescent women with age 13-19 years, others included 18-40 years (Asemi 2012; Asemi 2013; Mallet 1986; Roth 2013) and for remaining there was no data on age.  Intervention and control: Control group received either placebo or no intervention. Three RCTs provided D2: 800 IU/day (Yu 2008); 1000 IU/day (Brooke 1980); and in one arm 1000 IU/d and 200,000 IU single dose in another arm (Mallet 1986). Two RCTs provided D3 200 IU/d and calcium 500mg/d (Asemi 2012; Diogenes 2013). One RCT provided D3 50,000 IU/d, 400 microgram iron and folic acid 60 mg/d (Asemi 2013). One RCT provided D3 1000 IU/d (Delvin 1886), one RCT provided 1000 IU/d in one arm and 2000 IU/d in another arm (Grant 2013), one RCT provided 35,000 IU/week (Roth 2013) and one RCT provided 60,000 or 120,000 IU of three or four doses in pregnancy (Sablok 2015). Follow-up period range from six weeks to 13 weeks and followed till term. Only one RCT used high performing liquid chromatography to assess the outcome (Roth 2013). Only one RCT had low risk based on all criteria of Cochrane ROB tool (Grant 2013), two (Asemi 2013; Asemi 2012) had unclear reporting bias, one (Roth 2013) had unknown other bias, and rest all had more than one ROB criteria.  Meta-analysis was performed using seven RCTs (Asemi 2013; Brooke 1980; Delvin 1986; Grant 2013; Mallet 1986; Roth 2013; Sablok 2015) with 507 women in intervention and 361 in control group. It was found that supplementation with Vitamin D significantly increased the 25(OH)D levels compared to no intervention or placebo. Mean difference using random effect measure (REM) was 54.73 (95%CI: 36.60, 72.86) nmol/L with high heterogeneity (I^2^: 99%) and SR authors concluded that results would be interpreted with caution. Additionally, based on GRADE, authors concluded that evidence for ‘25(OH)D concentration level’ outcome is low level of evidence. Authors stated that they cannot determine the clinical implication of vitamin D supplementation during routine antenatal care. Subgroup analysis was conducted and reported that the effect was beneficial but had high heterogeneity, subgroups had one or two trials with misleading results. Pregnant women who received vitamin D daily had higher concentration of 25(OH)D levels at term/delivery, but there were differences in dose and outcome assessment in included RCTs (Regil et al., 2016).  Result of sub group analysis is as follows;   1. Start of vitamin D supplementation: 2. < 20 weeks (Delvin 1986), n=15(I)/17(C): Mean difference (REM): 32.45 [95% CI: 19.48, 45.42] nmol/L (Regil et al., 2016). 3. 20/> weeks of pregnancy (Asemi 2013; Brooke 1980; Grant 2013; Mallet 1986; Roth 2013; Sablok 2015) with n=492(I)/344(C): Mean difference (REM): 49.70 [36.62, 62.78; I^2^: 99%] nmol/L (Regil et al., 2016). 4. Combined MD (REM): 47.24 [35.17, 59.31; I^2^: 99%] nmol/L, n: 507/361 (Regil et al., 2016). 5. Weight of the participants:   normal, 18.5-24.9 kg/m2 (Sablok 2015), n=108/57: MD: 34.09 [12.51, 55.67] nmol/L (Regil et al., 2016).  overweight, 25 or more kg/m2 (Asemi 2013; Grant 2013), n=197/111: 19.54 [18.34, 20.74; I^2^:89%] nmol/L (Regil et al., 2016).  mixed/unknown/unreported (Brooke 1980; Delvin 1986; Mallet 1986; Roth 2013), n=202/193, MD: 73.18 [21.00, 125.36; I^2^:99%] nmol/L (Regil et al., 2016).  Combined: MD (REM) 47.24 [35.17, 59.31, I^2^:99%] nmol/L, n=507/361 (Regil et al., 2016).  iii. Frequency of vitamin D:  single dose (Mallet 1986; Sablok 2015; Yu 2008), n=195/154, MD (REM): 15.16 [ 5.68, 24.63, I^2^: 81%] nmol/L (Regil et al., 2016).  daily dose (Asemi 2013; Brooke 1980; Delvin 1986; Grant 2013; Mallet 1986; Roth 2013), n=399/304, MD (REM): 57.80 [ 38.37, 77.23, I2:99%] nmol/L (Regil et al., 2016).  Combined MD (REM): 44.12 [30.24, 58.00, I^2^:99%] nmol/L, n=594/449 (Regil et al., 2016).  vi. Latitude:  Between Tropics of Cancer and Capricorn (Grant 2013), n=173/87, MD (REM):19.13 [17.79, 20.47] nmol/L (Regil et al., 2016).  North of the Tropic of Cancer or South of the Tropic of Capricorn (Asemi 2013; Brooke 1980; Delvin 1986; Mallet 1986; Roth 2013; Sablok 2015), n:334/274, MD (REM): 55.73 [35.67, 75.80, I^2^: 99%] nmol/L (Regil et al., 2016).  Combined MD (REM): 47.24 [35.17, 59.31, I^2^:99%] nmol/L, n=507/361 (Regil et al., 2016).   1. Season at the start of pregnancy:   Summer (Roth2010), n= 80/80, MD (REM): 96.00 [88.19, 103.81] nmol/L (Regil et al., 2016)  Winter (Mallet 1986), n=48/29, MD (REM): 16.30 [13.61, 18.99] nmol/L (Regil et al., 2016)  Mixed season (Asemi 2013; Brooke 1980; Delvin 1986; Grant 2013; Sablok 2015), n:379/252= MD (REM): 37.24 [27.46, 47.02, I^2^:96%] nmol/L (Regil et al., 2016)  Combined= MD (REM): 47.24 [35.17, 59.31, I^2^:99%] nmol/L, n=507/361 (Regil et al., 2016)  A good quality SR (Roth et al., 2017), included 32 trials having 42 comparisons with 5706 women, conducted a meta-analysis and found that intervention was effective to increase serum 25(OH)D levels at or near delivery (Weighted Mean Difference (WMD): 32.91 nmol/L, 95%CI 27.19, 38.62, I^2^ 96%). SR authors reported that, included trials published until 2017 were of low quality, small in size and data on outcome were heterogeneous, therefore it is difficult to conclude. Included trials were (Abotorabi 2017; Asemi 2013a and b; Brooke 1980; Chawes 2016; Cooper 2016; Dawodu 2013; Delvin 1986; Grant 2014; Hashemipour 2013; Hollis 2011; Karamali 2015; Khan 2016; Litonjua 2016; Mallet 1986; March 2015; Mojibian 2015; Mutlu 2014; Rodda 2015; Roth 2013; Sabet 2012; Sablok 2015; Sahu 2009; Sahoo 2016; Soheilykhah 2013; Thiele 2016; Valizadeh 2016; Vaziri 2016; Yap 2014; Yazdchi 2016; Yu 2009; Zerofsky 2014). Quality of included trials varied.  Authors (Roth et al., 2017) carried out sensitivity analysis and the effect was beneficial. Irrespective of the group, intervention was effective.  The result of sensitivity analysis are as follows:   1. Based on year of publishing (in and after 2000): 30 trials, 38 comparisons with n= 5469, WMD: 31. 59 (25.50, 37.68) nmol/L, I^2^: 95.4% (Roth et al., 2017). 2. Based on quality of the trials (low): 8 trials, 9 comparisons with n=2109, WMD: 43.09 (29.69, 56.49) nmol/L, I^2^: 97.1% (Roth et al., 2017). 3. Based on health status of the women (with no pre-existing health condition) 28 trials and 38 comparisons with n=5411, WMD: 31.72 (25.77, 37.67) nmol/L, I^2^: 95.9% (Roth et al., 2017).   The result of subgroup analysis are as follows:  Based on type of control. Placebo control: 18 trials with 22 comparisons, with n=2600, WMD: 37.10 (28.57, 45.64) nmol/L, I^2^: 96.8%. Active control: 14 trials, 20 comparisons with n=3106, WMD: 28.76 (21.14, 36.38) nmol/L, I^2^: 93.4% (Roth et al., 2017).  Based on intervention (D_2_/_3_). Vitamin D_2_: 3 trials, 4 comparisons with n=322, WMD: 41.91 (23.43, 60.39) nmol/L, I^2^:97.3. Vitamin D_3_: 29 trials, 36 comparisons with n=5330, WMD: 31.97 (25.59, 38.34) nmol/L, I^2^: 95.8% (Roth et al., 2017).  Based on dose. Regular dose: 28 trials and 35 comparisons with n= 5232, WMD: 34.63 (28.23, 41.03) nmol/L, I^2^: 95.8%. Bolus dose: 6 trials, 7 comparisons with n=562, WMD: 21.74 (13.19, 30.29) nmol/L, I^2^: 87.5% (Roth et al., 2017).  Based on region. Europe: 7 trials, 10 comparisons with n=2045, WMD: 26.50 (18.05, 34.95) nmol/L, I^2^:95.6%. South Asia: 4 trials, 6 comparisons with n=431, WMD: 39.23 (6.76, 71.70) nmol/L, I^2^:97.4%. Americas: 5 trials, 7 comparisons with n=1410, WMD: 21.54 (12.78, 30.30) nmol/L, I^2^:89.6%. Eastern Mediterranean: 13 trials, 15 comparisons with n=1389, WMD: 37.25 (26.80, 47.70) nmol/L, I^2^:94.4%. Western Pacific: 3 trials, 4 comparisons with n=431, WMD: 38.73 (29.81, 47.64) nmol/L, I^2^: 61.8% (Roth et al., 2017).  Based on period of initiation of the intervention followed up until term or delivery: Started in 1st trimester: 8 trials, 13 comparisons with n=2099, WMD: 22.86 (16.00, 29.72) nmol/L, I^2^:86.3%. Started in 2nd trimester: 11 trials, 13 comparisons, n= 2562, WMD: 42.24 (29.34, 55.14) nmol/L, I^2^:97.1%. Started in 3rd trimester: 3 trials, 3 comparisons, n=189, WMD: 59.37 (23.34, 95.39) nmol/L, I^2^: 98.2% (Roth et al., 2017).  Based on maternal baseline levels of 25OHD levels. <30 nmol/L: 7 trials, 12 comparisons, n=791, WMD: 32.87 (19.47, 46.27) nmol/L, I^2^: 95.2%. ≥30 nmol/L: 22 trials, 26 comparisons, n=4791, WMD: 35.20 (27.69, 42.70) nmol/L, I^2^: 95.9% (Roth et al., 2017).  Based on effective daily equivalent dose considering regular dose trials. <2000 IU/day: 14 trials, 17 comparisons, n=2307, WMD: 25.74 (19.19, 32.29) nmol/L, I^2^: 91.9%. ≥2000 IU/day: 18 trials, 18 comparisons, n=3191, WMD: 41.78 (32.46, 51.10) nmol/L, I^2^: 96% (Roth et al., 2017).  Result of meta-regression of the WMD are as follows:  i. Baseline maternal 25(OH) D concentration: the estimated change in maternal 25(OH)D at delivery for every 20 nmol/L increase was 5.1 nmol/L (Roth et al., 2017).  ii. Maternal 25(OH)D concentration of the control group at delivery: the estimated change in maternal 25(OH)D at delivery for every 20 nmol/L increased was 1.8 nmol/L (Roth et al., 2017).  iii. Effective dose: the estimated change in maternal 25(OH)D at delivery for every 400 IU/day increase in effective dose was 3.0 nmol/L (Roth et al., 2017).  A moderate quality SR (Chakhtoura et al., 2017) pooled the result based on dose of vitamin D and performed the following comparison;   1. High v/s intermediate and intermediate v/s low dose: Three arm RCTs conducted among pregnant women in UAE with unclear ROB on all the domains of Cochrane ROB tool (Dawodu 2013) and conducted in Iran with high risk of performance and selection bias (Soheilykhah 2013). Chakhtoura et al., 2017 summarised that high v/s intermediate and intermediate v/s low dose were beneficial in increasing 25(OH)D levels in women. Vitamin D/day was started in 12-16 weeks of pregnancy and followed until delivery. Dawodu 2013 provided D_3_, 3600 IU/d in one arm, 1600 IU/d in second and placebo in third. Soheilykhah 2013 provided D_2_, 50,000 IU every 2 weeks, 50,000 IU per month and 200 IU/day in three arms, respectively. Mean ages were reported as 25.6-27.5 (Dawodu 2013) and 25-26.5 (Soheilykhah 2013). To measure the outcome none used high performing liquid chromatography. Intermediate dose v/s low dose: MD using REM was 7.82 nmol/L (95% CI: 4.84, 10.80, I^2^: 0%) using n=79/77 in intermediate/low dose groups. For high dose v/s intermediate dose: MD using REM was 8.61 nmol/L (95% CI: 5.32, 11.91, I^2^: 0%) and included n=83/79 in high/intermediate dose group (Chakhtoura et al., 2017). 2. High dose of vitamin D (n=204) v/s low dose (n=201) was assessed using five RCTs. One RCT was from UAE (Dawodu 2013) and others from Iran (Soheilykhah 2013; Karamali 2015; Samimi 2015; Vaziri 2016). Two RCTs had included pregnant women having preeclampsia (Karamali 2015; Samimi 2015) and remaining three included healthy pregnant women. Mean age range was 25-27.3 years and mean BMI ranged from 25- 27.4 kg/m2. Outcome assessment varied in all five RCTs. Intervention varied in all RCTs; one (Soheilkhan 2013) used D_2_ 50,000 IU/m or every two weeks in two different arms and remaining others used D_3_ 1600-3600 IU/d, or 50,000 IU/ 2 weeks. Start of intervention was second trimester continued till term or 32 weeks of gestation. Control group was 200-400 IU/d along with multivitamins or placebo. MD using REM was 12.3 (95%CI 6.4, 18.2, I^2^ 95%) ng/ml, favouring high dose. Sensitivity analysis using only low ROB RCTs (Dawodu 2013; Karamali 2015; Samimi 2016) found a MD using REM of 13.1 (95% CI 5.4, 20.7) ng/ml, favouring the high dose (Chakhtoura et al., 2017). 3. High dose v/s placebo: Chakhtoura et al., 2017 included two RCTs. One RCT (Etemadifar 2015) included pregnant women with multiple sclerosis and other included health pregnant women (Sabet 2012). Mean ages of women were 26-30 years. Intervention D_3_ 50,000 IU/week (Etemadifar 2015) and 100,000 IU/month (Sabet 2012). Start of intervention 12-16 (Etemadifar 2015) and 27 (Sabet 2012) weeks of gestation. Meta-analysis was not possible as outcome measurements were undertaken at two different time points, one at delivery (Sabet 2012) and one six months post-delivery(Etemadifar 2015) |
| 2.1.2. Vitamin D deficiency symptoms: |
| The evidence for the effect of vitamin D supplementation on vitamin D deficiency symptoms was not sufficient, due to only one low quality RCT (Marya 1988) (as specified by the authors of SR with no details on tool that was used) conducted in India, in a moderate methodological quality SR (Christesen et al., 2012). Pregnant women (n=200), 22-35 years, intervention group received 600,000 IU D_3_ twice at seven and eighth month compared to placebo. The SR concluded that intervention was effective. |
| 2.1.3. Bone mineral density (BMD): |
| There was lack of sufficient evidence for the effect of vitamin D supplementation on BMD as only one RCT (Islam et al., 2010) was identified in a moderate methodological quality SR (Reid, Bolland & Grey, 2014). Quality of included RCTs was measured using Cochrane tool and there was unclear description of randomisation and allocation concealment. Intervention details were; D_3_ 400 IU/d for 12 months v/s placebo on adult women from Bangladesh (n=100) with mean age 22 years. 25OHD significantly increased during the study period in the vitamin D group. Weighted mean differences: in lumbar spine BMD (%): 1·7 (–0·5 to 3·9) nmol/L. WMD in femoral neck BMD (%): 2·8 (1·5 to 4·1) nmol/L. WMD in total hip /trochanter BMD: 3·0 (1·2 to 4·8) nmol/L. These findings might be due to chance as SR authors assume that Bangladeshi women might have had low intake of dietary calcium and might be more deficient in vitamin D than the measurements suggest. |
| 2.1.4. Serum calcium levels: |
| Result of supplementation with vitamin D on serum calcium levels was reported in a moderate methodological SR (Chakhtoura et al., 2017) including three arm RCTs conducted among pregnant women in UAE with unclear ROB on all the domains of Cochrane ROB tool (Dawodu 2013) and conducted in Iran with high risk of performance and selection bias (Soheilykhah 2013). Vitamin D/day was started in 12-16 weeks of pregnancy and followed until delivery. Dawodu 2013 provided D_3_, 3600 IU/d in one arm, 1600 IU/d in second and placebo in third. Soheilykhah 2013 provided D_2_, 50,000 IU every 2 weeks, 50,000 IU per month and 200 IU/day in three arms, respectively. Mean ages were reported as 25.6-27.5 (Dawodu 2013) and 25-26.5 (Soheilykhah 2013).  Meta-analysis result- High v/s intermediate dose: MD: 0.06 (-0.06, 0.18) mg/dl and intermediate v/s low dose: MD: -0.05 (-0.41, 0.30) mg/dl. |
| 2.1.5. Weight gain |
| Vitamin D supplementation did not have a significant effect on daily weight gain as there was only one low quality RCT (Mayra et al., 1988) (as specified by the authors of SR with no details on tool that was used) conducted in India, in a moderate methodological quality SR (Christesen et al., 2012). Pregnant women (n=200), intervention group received 600,000 IU D_3_ twice at 7 and 8^th^ month compared to placebo and concluded that intervention was effective. |
| 2.1.6. Side effects of vitamin D: |
| 1. Nephritic syndrome: Only one RCT (Yu 2008) was identified from a good methodological quality SR (Regil et al., 2016). Participants included were pregnant women. Group 1 (n=60) received a daily dose of vitamin D (ergocalciferol D2) at 800 IU/d, group 2 (n = 60) received a stat dose of 200,000 IU of calciferol; group 3 (n = 60) received no treatment. Duration of the intervention was 13 weeks from start of supplementation to delivery. SR authors stated they cannot conclude as there was lack of data and wider confidence interval. Risk of nephritic syndrome was same among women who received vitamin D supplementation compared to women who did not receive intervention or placebo. Authors further reported that there was only single case of nephritic syndrome occurred in women with no supplementation. As per the Cochrane ROB tool, there was high risk on performance bias, unclear on detection and other bias. 2. Hypercalcemia:    - 1. One robust SR (Roth et al., 2017) demonstrated insignificant (n= 175, RR: 3.11, CI: 0.87, 11.08) result of vitamin D supplementation on hypercalcemia, four RCTs were included, however, only one (Hossain 2014) contributed towards the outcome.      2. SR (Roth et al., 2017) further included trials without applying for case definition or method of ascertainment of outcome. Nine trials measured the hypercalcemia but, only two trials (March 2015; Hossain 2014) with three comparisons were eligible. Pooled RR with REM: 1.84 (0.84, 4.02), I^2^= 0%.      3. Authors (Roth et al., 2017) tried performing sensitivity and subgroup analysis but the result was not significant and the events in eligible trials were not identified or less in number. Other details are not available. 3. Hypercalciuria: 4. Two trials reported the outcome but one identified the event (Roth 2013) from one SR (Roth et al., 2017). Trial included 160 participants and the result was insignificant (RR: 3, CI: 0.12, 72.56). 5. SR (Roth et al., 2017) further included trials without applying for case definition or method of ascertainment of outcome and identified two trials (March 2015; Roth 2013) with three comparisons. The result was insignificant (Pooled RR: 1.12, CI: 0.63, 1.99, I^2^: 0%). Other details are not available.   iv, Hypocalcaemia:   1. Two trials reported the outcome but one identified the event (Marya 1988) from one SR (Roth et al., 2017). Trial included 200 participants and the result was significant favouring intervention (RR: 0.05, CI: 0.01, 0.18). 2. SR (Roth et al., 2017) included trials without applying case definition or method of ascertainment of outcome and identified two trails (Marya 1988; Hashemipour 2013). Intervention had preventive effect on maternal hypocalcaemia (Pooled RR: 0.04, CI: 0.01, 0.14, I^2^: 0%). Other details are not available. |
| 2.2. Vitamin D fortification |
| 2.2.1.Serum Vitamin D levels |
| Insufficient information of fortification of vitamin D on serum vitamin D levels as there was only one included RCT (Green 2010) involving 66 participants. RCT was conducted in New Zealand with women of age ranging from18-47 years. Targeted fortification strategy was used to fortify milk with 5 gram of vitamin D for 12 weeks. The result was insignificant (SMD: 0.26 (95% CI: -0.22, 0.75) and level of evidence as assessed by GRADE was moderate. The evidence is from moderate methodological SR (Das et al., 2013) and as per Cochrane ROB tool only attrition rates and blinding was adequately discussed. |
| 2.2.2. Serum calcium levels |
| A moderate quality SR (Das et al., 2013) included one trial (Natri 2006) with two groups Rye and Wheat involving 39 women of reproductive age (25-45 years) from Denmark. Bread was fortified with 10 micro grams/100 gms of vitamin D for three weeks. Standardized Mean Difference using REM was -0.18 (-2.04, 1.69, I^2^: 87%). As per Cochrane ROB tool, only attrition rates were adequately discussed in RCT and SR scored moderate on RMASTAR. |
| 2.3. Vitamin D and calcium fortification |
| 2.3.1. Serum vitamin D levels |
| Insufficient information for vitamin D and calcium fortification on serum vitamin D levels as there was only one included RCT (Kruger 2006) involving 55 participants. RCT was conducted among women age 20-35 years from New Zealand. Skimmed milk was fortified with 1000 mg of calcium and 5 g of vitamin D3. Targeted fortification was followed for 16 weeks. The result was significant (SMD: -2.50 (95% CI: -3.22, -1.78) and level of evidence was moderate as per GRADE. The evidence is from moderate methodological SR (Das et al., 2013) and as per Cochrane ROB tool only attrition rates and blinding was described adequately. |
| 2.3.2. Serum calcium levels |
| The result favoured fortification group but insufficient to conclude as only one RCT (Kruger 2006) involving 55 participants contributed towards the outcome. RCT was conducted among women age 20-35 years from New Zealand. Skimmed milk was fortified with 1000 mg of calcium and 5 g of vitamin D3. Targeted fortification was followed for 16 weeks. The evidence is generated from moderate quality SR (Das et al., 2013) and as per Cochrane ROB tool only attrition rates and blinding was described adequately. |
| 2.3.3.CTx and P1NP (bone resorption marker) levels |
| Result of P1NP and CTX (bone resorption marker) levels favoured the fortified group, however, there was only one included trial (Kruger 2006) involving 55 women of reproductive age. RCT was conducted among women age 20-35 years from New Zealand. Skimmed milk was fortified with 1000 mg of calcium and 5 g of vitamin D3. Targeted fortification was followed for 16 weeks. The evidence is generated from moderate quality SR (Das et al., 2013) and as per Cochrane ROB tool only attrition rates and blinding was described adequately. |
| 3. Calcium supplementation |
| 3.1. Maternal Body weight and weight gain |
| Three SRs of high (Buppasiri, et al., 2016) and moderate (Trowman, et al., 2006; Onakpoya, et al., 2011) methodological quality were included.  A moderate methodological quality SR (Trowman et al., 2006) included one RCT (Winter-Stone & Snow, 2004) conducted in USA among athletes with mean age 24.8 years. Supplementation of calcium 1000 mg/d followed-up till 12 months (n=13) was compared to placebo group (n=10). There is lack of sufficient evidence to conclude the effect of calcium supplementation on body weight compared to placebo.  Another moderate methodological quality SR (Onakpoya et al., 2011), had RCTs conducted in USA (Ricci 1998; Riedt 2005; Riedt 2007; Shapses 2001; Shapses 2004; Yanovski 2009) and Iran (Shalileh 2010). The analysis data for only three RCTs i.e. Riedt 2007; Shapses 2001; Shapses 2004, which were on premenopausal women is provided in this review.The RCTs were of heterogeneous quality and had obese and overweight women as participants with mean age range 36.6 to 42.1 years. Intervention consisted of 1000 mg calcium tablets to premenopausal women for six months compared to placebo. Riedt 2007 applied control on lifestyle factors (responsible for weight gain), viz. 1200-1500 kcal daily intake, 600 mg dietary Ca daily; Shapses 2004 applied lifestyle factor control such as normal lifestyle, 2100 KJ deficit daily, 700 mg dietary calcium daily, and Shapses 2001 applied lifestyle control factors such as normal lifestyle, 1263-1281 kcal daily and 810-1005 dietary calcium daily.  Meta-analysis including three RCTs with 190 participants reported that the weight loss in calcium supplemented group was significantly more when compared to the placebo group i.e. MD -1.32 kg (95% CI: -2.58 to -0.06, I^2^: 0%) (Onakpoya et al., 2011).  High quality SR (Buppasiri, et al., 2015) assessed calcium supplementation and its effect on maternal weight gain. The SR included three RCTs (Villar 1987; Lopez-Jaramillo 1997; Lopez-Jaramillo 1989) with 404 pregnant women, number ranged from 25(I)/27(C) (Villar 1987) to 125(I)/135(C) (Lopez-Jaramillo 1997). Pregnant women had resided at an altitude of 2800 meters in Ecuador, were under 17.5 years (Lopez-Jaramillo 1997); Ecuador, age less than 25 years (Lopez-Jaramillo 1989); and Argentina, age 18-30 years (Villar 1987).  Dose: 1000 mg or more calcium supplementation started at 20 weeks of pregnancy (Lopez-Jaramillo 1989), three tablets of calcium carbonate 500 mg each, recruited at 26 weeks of pregnancy (Villar 1987) and 2000 mg calcium (four tablets of calcium carbonate per day having 500 mg of elemental calcium) with unclear starting time (Lopez-Jaramillo 1997).  Two RCTs (Lopez-Jaramillo 1997; Villar 1987) scored low risk for all the domains in the Cochrane ROB tool whereas one RCT (Lopez-Jaramillo 1989) scored low risk for five domains and unclear risk for attrition and reporting bias. It was reported that 2000 mg of daily calcium supplements to pregnant women has no significant effect on the maternal weight gain when compared to placebo MD -29.46 grams per week (95% CI -119.80 to 60.89 g per week, I2=80%) (Buppasiri, et al., 2015).  Overall the evidence from these reviews suggests that supplementation of calcium for minimum duration of six months significantly resulted in weight loss in overweight and obese individuals, but there was no effect of calcium supplementation on body weight of pregnant women. The clinical relevance of this finding is uncertain. The review recommends future research to be undertaken for well-conducted RCTs for more appropriate conclusion. |
| 3.2. Bone Mass/BMD |
| The information is generated from low quality (Cumming, 1990) and good quality (Buppasiri et al., 2015; Arthur et al., 2015) SRs. All three SRs included one RCT in each (Raman 1978; Smith 1989; Jarjou 2010), and each of these RCTs provided contradicting results, hence there was not much information to conclude the effect of calcium supplementation on bone mass or BMD.  Buppasiri et al., 2015 assessed maternal bone mineral density that was reported by one high ROB RCT (Raman 1978) with 273 pregnant women from low socio-economic status in India. This was a three arm trial with one group received 300 mg/day of calcium (n=25), other group received 600 mg/day of calcium (n=24) and third control group received no intervention (n=38). Calcium lactate was given in tablet form with 150 mg of elemental calcium per tablet starting from 18 to 22 weeks until delivery. There was 68.1 % loss to follow up. In either of the dosages, the difference was not statistically significant between the intervention and control group.  Outcome was measured by X-ray left hand (anteroposterior view) and Densitometry of metacarpal and 4-1 phalangeal. Effect of Calcium on BMD [Maternal bone mineral density (g/cm2) – measured at first phalanx, second & fourth metacarpal in both calcium 300 mg and 600 mg] was insignificant (Buppasiri et al., 2015).  For the calcium dose of 300 mg (Buppasiri et al., 2015):   1. first phalanx: MD -0.07 g/cm2, 95% CI -0.29 g/cm2 to 0.15 g/cm2. 2. second metacarpal: MD 0.19 g/cm2, 95% CI -0.02 to 0.40 g/cm2. 3. fourth metacarpal: MD 0.06 g/cm2, 95% CI -0.17 to 0.29 gm/cm2.   For the calcium dose of 600 mg (Buppasiri et al., 2015):   1. first phalanx: MD 0.09 g/cm2, 95% CI -0.10 to 0.28 gm/cm2. 2. second metacarpal: MD 0.14 g/cm2, 95% CI -0.11 to 0.39 g/cm2. 3. fourth metacarpal: MD 0.07 g/cm2, 95% CI -0.13 to 0.27 g/cm2   Cumming 1990 included one RCT (Smith 1989) that provided 1500 mg calcium to 35 premenopausal women with 42 years as mean age. There was 0.02 mean difference bone mass on all bone sites indicating a protective effect of calcium compared to control.  Arthur et al., 2015 suggests that, calcium supplementation has negative effect on bone mineral density. A trial from Gambia (Jarjou 2010) provided 1500 mg of calcium per day (three calcium carbonate tablets) from 20 weeks of pregnancy until delivery. It was reported that calcium supplementation could be harmful as pregnant women given calcium supplements were found to “have lower bone mineral content, bone area, and bone mineral density at the hip during the 12-month lactation period” than the placebo group. The authors concluded that calcium supplementation may have interruption on metabolic adaptation |
| 3.3. Anaemia |
| A good quality SR (Buppasiri, et al., 2015) including one good quality RCT (Belizan 1991) reported maternal anaemia. The RCT scored low risk for all the domains in the Cochrane ROB tool and included 1098 (593 in treatment and 601 in control group) nulliparous pregnant women from Argentina. It was reported that supplementation of 2 g calcium, (4 tablets/day; each calcium tablet contained 500 mg calcium carbonate and granulated starch), started at 20 weeks of gestation.  The effect of calcium supplementation on maternal anaemia was not significant; when compared to the controls RR 1.04 (95% CI 0.9 to 1.22). However, this result cannot be used to conclude the effect of calcium supplementation on maternal anaemia on the basis of one RCT. |
| - 1. Side effects of calcium supplementation |
| One high quality SR (Buppasiri, et al., 2015) with four RCTs having heterogeneous quality (Belizan 1991; Villar 1987; Villar 2006; Wanchu 2001) reported this outcome. The side effects reported include maternal cholestasis jaundice (Wanchu 2001), gastrointestinal symptoms consisting of diarrhoea, nausea and heartburn (Villar 1987), gall stones (Belizan 1991) and multiple symptoms (Villar 2006). All the RCTs included pregnant women as the participants.  Villar 1987 provided three tablets of calcium carbonate (50 mg each) starting at 26 weeks of gestation until delivery, to the treatment group and three placebo tablets to the control group. Villar 2006 provided 1.5 gms of calcium carbonate (1*500 mg tablets, three times per day at meal times), starting at 20 weeks of gestation until delivery, and three tablets of placebo (sorbitol, lactose, cellulose plus other calcium free ingredients) per day. Wanchu 2001 provided 2 gms of calcium (four tablets of calcium carbonate) starting at 20 weeks of gestation, compared with no treatment. Belizan 1991 provided 2 g calcium/d (4 tablets/day; each calcium tablet contained 500 mg calcium carbonate and granulated starch), started at 20 weeks of gestation, compared with placebo.  For maternal cholestasis jaundice, there was no statistically significant difference between the groups, as was reported by one trial that included 100 women (Wanchu 2001) (RR 3.00, 95% CI 0.13 to 71.92). No statistically significant difference between the groups for gastrointestinal symptoms reported by one trial involving 52 women (Villar 1987) (RR 2.16, 95% CI 0.43 to 10.78). No statistically significant difference for Gall stones between the groups reported by one trial involving 518 women (Belizan 1991) (RR 1.35, 95% CI 0.48 to 3.85). The results were not statistically significant for the groups that reported for headache, vomiting, backache, vaginal and urinary complaints, swelling, dyspepsia, abdominal pain reported by one trial involving 8312 women (Villar 2006) (RR 1.02, 95% CI 0.93 to 1.12).  Overall, no statistically significant difference between the intervention and control groups for the side effects of calcium was reported. However, this result is not sufficient for conclusion due to non-availability of adequate evidence. |
